# Genome-wide characterization and comparative analysis of the OSCA gene family and identification of its potential stress-responsive members in legumes

**DOI:** 10.1038/s41598-023-33226-8

**Published:** 2023-04-11

**Authors:** Srija Chakraborty, Rashmi Gangwar, Shafaque Zahra, Nikita Poddar, Amarjeet Singh, Shailesh Kumar

**Affiliations:** 1 Bioinformatics Lab, National Institute of Plant Genome Research (NIPGR), Aruna Asaf Ali Marg, New Delhi, 110067 India; 2 Stress Signalling Lab, National Institute of Plant Genome Research (NIPGR), Aruna Asaf Ali Marg, New Delhi, 110067 India

**Keywords:** Data mining, Data processing, Gene expression, Gene regulation, Bioinformatics

## Abstract

*Cicer arietinum*, *Cajanus cajan*, *Vigna radiata*, and *Phaseolus vulgaris* are economically important legume crops with high nutritional value. They are negatively impacted globally by different biotic and abiotic stresses. Hyperosmolality-gated calcium-permeable channels (OSCA) have been characterized as osmosensors in *Arabidopsis thaliana* but have not previously reported in legumes. This study provides a genome-wide identification, characterization, and comparative analysis of *OSCA* genes in legumes. Our study identified and characterized 13 *OSCA* genes in *C. cajan*, *V. radiata*, *P. vulgaris,* and 12 in *C. arietinum*, classified into four distinct clades. We found evidence to suggest that the *OSCAs* might be involved in the interaction between hormone signalling pathways and stress signalling pathways. Furthermore, they play a major role in plant growth and development. The expression levels of the *OSCAs* vary under different stress conditions in a tissue-specific manner. Our study can be used to develop a detailed understanding of stress regulatory mechanisms of the *OSCA* gene family in legumes.

## Introduction

Plants are continuously subjected to multiple stress stimuli and steadily adapt to complex environmental challenges. Among numerous abiotic stresses, drought and salinity stress resulting in osmotic stress is a major constraint in plant growth and development owing to cell membrane damage, phosphorus deficiency, etc. It significantly impairs the metabolic pathways of plants, such as photosynthesis, respiration, mineral assimilation, and biomass accumulation^1^, which leads to food shortages that adversely affect society.

Drought tolerance is a multifaceted and intrinsic trait. Food crop association and candidate gene studies are intensely directed toward selecting favourable alleles for drought resistance. Various allelic variations have been discovered that facilitate drought tolerance^1^. As ideally sessile organisms, plants have devised several mechanisms, such as changes in physiological and morphological adaptations, to bypass these conditions to survive.

Calcium-permeable channels potentially function as osmosensors in bacteria and animals under osmotic and mechanical stress^2^. They perceive external osmotic stress and consequently activate multiple signal transduction pathways. In most plant signalling pathways, Ca^2+^ acts as a versatile second messenger system^3^. When triggered by hyperosmolality stimuli, these osmosensitive channels are opened, giving rise to a rapid elevation of cytosolic concentration of free calcium ions, which is a primary response to osmotic stress. In plants, hyperosmolarity-evoked intracellular calcium increase is regulated by the OSCA gene family^4^. The OSCA (hyperosmolarity-gated calcium-permeable channel) is a calcium nonselective cation channel protein that also acts as a receptor protein for hypertonic stress^5^. It serves as mechanosensitive-ion channels/osmosensors playing a crucial role in regulating osmotic stress in plants. In *Arabidopsis*, 16 genes were discovered in plants under drought stress, named Early Response to Dehydration (ERD), among which ERD4 contained a highly conserved domain called the DUF221 domain (Pfam accession: 02714)^1,6^. OSCA1 has been identified as an osmosensor in *Arabidopsis*^5^, which also consists of a DUF221 domain, indicating that the OSCA gene family participates in osmotic regulation. The stomatal aperture was increased in osca1 mutants during hyperosmotic treatments, and detached leaves of these mutants lose water much more rapidly than wild-type controls^5,7^. The presence of the DUF221 domain is also evident in the rice OSCA gene family^6^.

The Leguminosae family members play a vital role in agriculture and are potential sources of human nutrition, and more importantly, have a vital role in nitrogen fixation. Among the legume crops, chickpea (*Cicer arietinum*), pigeonpea (*Cajanus cajan*), mungbean (*Vigna radiata*), and common bean (*Phaseolus vulgaris*) provide nutritional support for the underprivileged and largely malnourished populations of the semi-arid tropics. Chickpea, native to South Asia and Sub-saharan Africa, is a highly nutritious crop with high protein (24.6%) and carbohydrate (64.6%) content, and a good source of minerals and fibers. Abiotic stresses are responsible for 40–60% of global annual chickpea production losses. About 50% loss of chickpea yield is due to drought, while cold and salinity stresses also act as a major limiting factor^8^. Additionally, it is also subjected to *Helicoverpa* and *Ascochyta* blight^9^. Mung bean (*Vigna radiata* (L.) R. Wilczek) contains a wide range of adaptability and high-stress tolerance and is a rich source of folate, iron, and high-quality proteins. Production of mung bean is greatly hampered by drought conditions and biotic constraints like Phytophthora infection, *Cercospora* leaf spot (CLS) etc^10,11^. *Cajanus cajan* is a climate resilience crop, although its yield is adversely affected by *Fusarium udum* pathogen attack and sterility mosaic disease^12^. Common bean (*Phaseolus vulgaris*) is another essential legume, with seeds enriched with lectin and α-Amylase inhibitors (α-AI). Drought severely affects as much as 60% of common bean production, and phosphorus constraint can affect almost 50% of common bean production^13^. Hence, the loss of productivity caused by various biotic and abiotic stresses leaves a huge gap between the demand and supply of these legumes.

The OSCA gene family has been an active research topic for many years due to its pivotal role in osmotic stress mediation in plants. Earlier, studies were conducted in monocots like rice and dicots like pear and cotton^14^, but they are not widely studied in legumes. Researchers have substantially focussed on legumes because of their unique molecular composition and positive environmental impacts. Leguminous plants suffer tremendously from drought stress, resulting in reduced yield and adverse effects on nutritional quality. Therefore, it is critical to determine how these plants tolerate drought and other abiotic and biotic stresses.

Here, we have systemically identified and performed genome-wide analysis and expression profiling of the *OSCA* gene family in the four leguminous plants. A total of 13 genes were identified in each of the *Cajanus cajan* (pigeon pea), *Vigna radiata* (mung bean), *Phaseolus vulagris* (common bean), and 12 genes in *Cicer arietinum* (chickpea) genomes. The gene and protein structures, phylogenetic distribution, subcellular localization, and cis-regulatory elements in the promoters of these genes were extensively characterized. Furthermore, global expression analysis was performed under biotic and abiotic stress conditions and during different stages of plant development. We have also validated the expression of *CaOSCA* genes through experimental techniques under salt, cold, and desiccation stress.

## Results

### Identification and sequence analysis of *OSCA* genes

After the BLASTp homology search, 19, 22, 20, and 13 putative OSCA proteins were found in *Cicer arietinum, Cajanus cajan, Vigna radiata,* and *Phaseolus vulgaris,* respectively. After removing redundant sequences and isoforms and confirming essential domains by InterProScan, 12 non-redundant OSCA proteins were identified in *C. arietinum,* and 13 non-redundant OSCA proteins in each of *C. cajan, V. radiata,* and *P. vulgaris* (Supplementary Table 1). The OSCA genes in each legume were named according to their homology with the previously identified OSCA proteins in *Arabidopsis* in each clade (*CaOSCA1.1* to *CaOSCA4.1, CcOSCA1.1* to *CcOSCA4.1, VrOSCA1.1* to *VrOSCA4.1, PvOSCA1.1* to *PvOSCA4.1*)*.* All three major protein domains, namely RSN1_TM (PF13967), PHM7_cyt (PF14703), and RSN1_7TM (PF02714), are found to be present in each OSCA protein sequence identified in all four plants in the current study, confirming the reliability of this methodology.

Clade IV proteins are the longest in all four legumes, ranging from 802 to 807 amino acids, and have the highest molecular weight (~ 90 kDa). Exceptionally, CaOSCA2.1 has the smallest length among all OSCA proteins in the study with a molecular weight of ~ 74 kDa, and VrOSCA1.5 has the highest molecular weight of 98.64 kDa and is 863 amino acids long (Supplementary Table 1). The length of the CaOSCA proteins ranges from 656 to 804 amino acids, with their molecular weights ranging from 73.95 to 91.8 kDa. In *C. cajan*, the length of the OSCA proteins ranged from 706 to 807 aa, and the molecular weight ranged between 80.3 and 92.4 kDa. In *V. radiata,* amino acid length and molecular weight vary between 710 and 863 amino acids and 80.8 to 98.6 kDa. The PvOSCA proteins are detected to be 711–853 aa long, with their molecular weights varying between 77 and 98 kDa (Supplementary Table 1). The theoretical isoelectric point (pI) of most OSCA proteins is above 7, indicating their basic nature. Interestingly, the clade IV OSCA proteins (CaOSCA4.1, CcOSCA4.1, VrOSCA4.1 and PvOSCA4.1) also exhibit significant deviation from the other three clades of their pI. Their isoelectric points are 7.48, 6.59, 6.59, and 6.69, respectively, in *C. arietinum, C. cajan, V. radiata* and *P. vulgaris*, suggesting that the clade IV proteins are neutral or mildly acidic in nature (Supplementary Table 1). Hence, most OSCAs, except OSCA4.1, are functional in similar alkaline sub-cellular surroundings. OSCA4.1 possibly requires a different microenvironment where it might be functional.

### Phylogenetic analysis of OSCA protein

**Figure 1:**
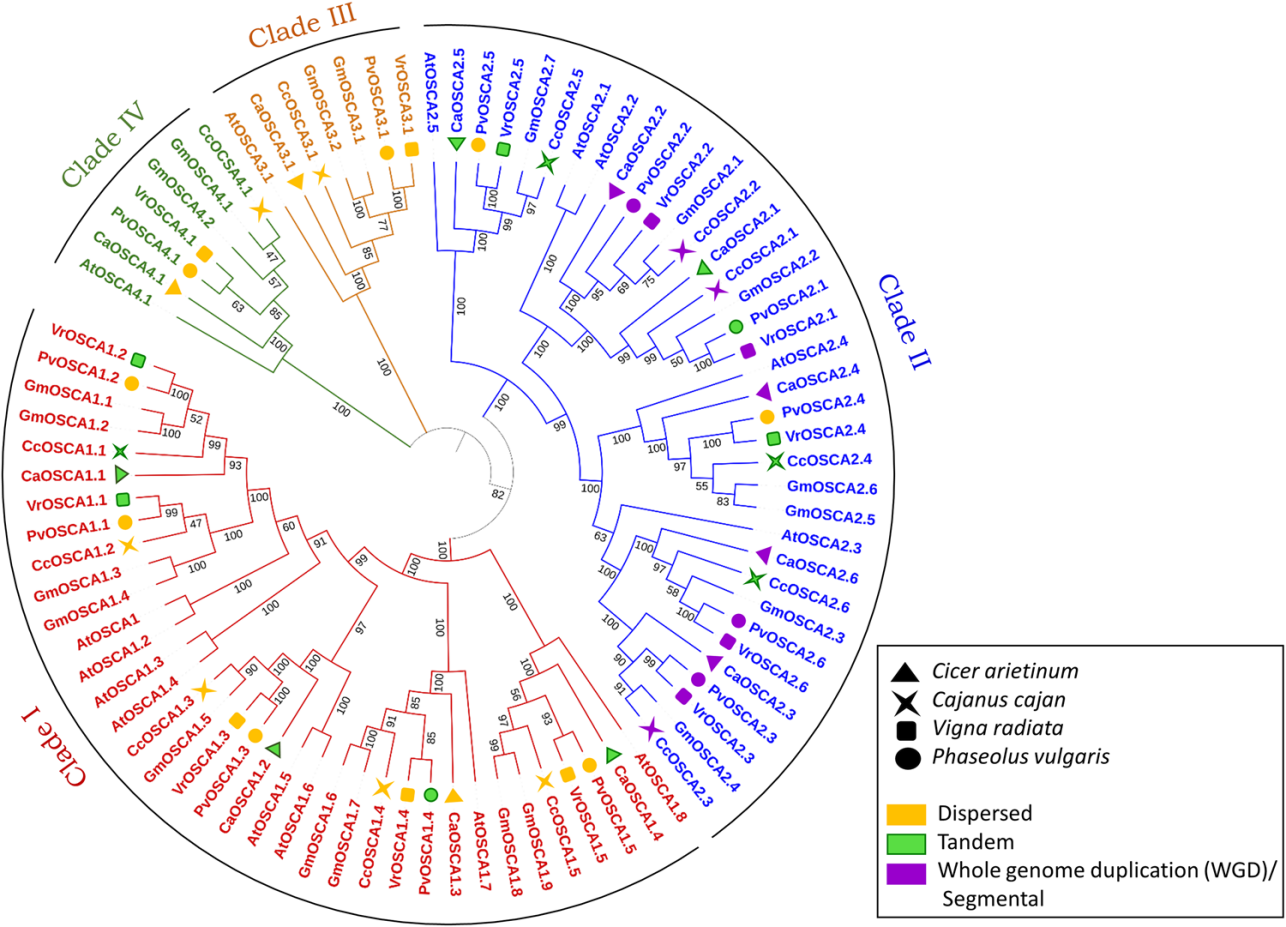
Phylogenetic relationship among OSCA from different species. The evolutionary relationship of OSCA proteins from *Arabidopsis thaliana* (At), *Glycine max* (Gm), *Phaseolus vulgaris* (Pv), *Cicer arietinum* (Ca), *Cajanus cajan* (Cc), and *Vigna radiata* (Vr) constructed using Neighbor-Joining method. Labels above the nodes represent bootstrap values calculated from 1000 replicates. Clades I, II, III and IV are represented by red, blue, green and orange lines respectively. The values in the phylogenetic tree represent bootstrap values. The gene duplication events behind the origin of each CaOSCA, CcOSCA, VrOSCA and PvOSCA is marked in the following colours: yellow- dispersed, green- tandem and purple- whole genome duplication/segmental.

To explore the phylogenetic relationship between the OSCA proteins in the legumes, a phylogenetic tree was constructed by using multiple sequence alignment data of 86 OSCA proteins of *Arabidopsis thaliana* (15), *Glycine max* (20) and 12 in *Cicer arietinum* and 13 in each of *Cajanus cajan*, *Vigna radiata* and *Phaseolus vulgaris* (Fig. 1). All 86 OSCA proteins were segregated into 4 distinct groups. Clades I and II are larger, and clades III and IV are comparatively smaller. Clade I contain 36 members consisting of eight members of *Arabidopsis* (AtOSCA1, -1.2, -1.3, -1.4, -1.5, -1.6, -1.7, and -1.8), nine members in *G. max* (GmOSCA1.1–1.9)*,* four members from *C. arietinum,* (CaOSCA1.1–1.4), and five members in each of *C. cajan*, *V. radiata* and *P. vulgaris* (CcOSCA1.1–1.5, VrOSCA1.1–1.5, PvOSCA1.1–1.5). Clade II also contains 36 members consisting of five members from *Arabidopsis* (AtOSCA2.1–2.5), seven members in *G. max* (GmOSCA2.1–2.7), six members each in *Cicer* (CaOSCA2.1–2.6), *Cajanus* (CcOSCA2.1–2.6), *Vigna* (VrOSCA2.1–2.6), *and Phaseolus* (PvOSCA2.1–2.6). Clade III and IV comprises of eight members each, AtOSCA3.1, GmOSCA3.1, GmOSCA3.2, CaOSCA3.1, CcOSCA3.1, VrOSCA3.1, PvOSCA3.1 and AtOSCA4.1, GmOSCA4.1, GmOSCA4.2, CaOSCA4.1, CcOSCA4.1, VrOSCA4.1, PvOSCA4.1 respectively.

### Gene and domain structure analysis

**Figure 2:**
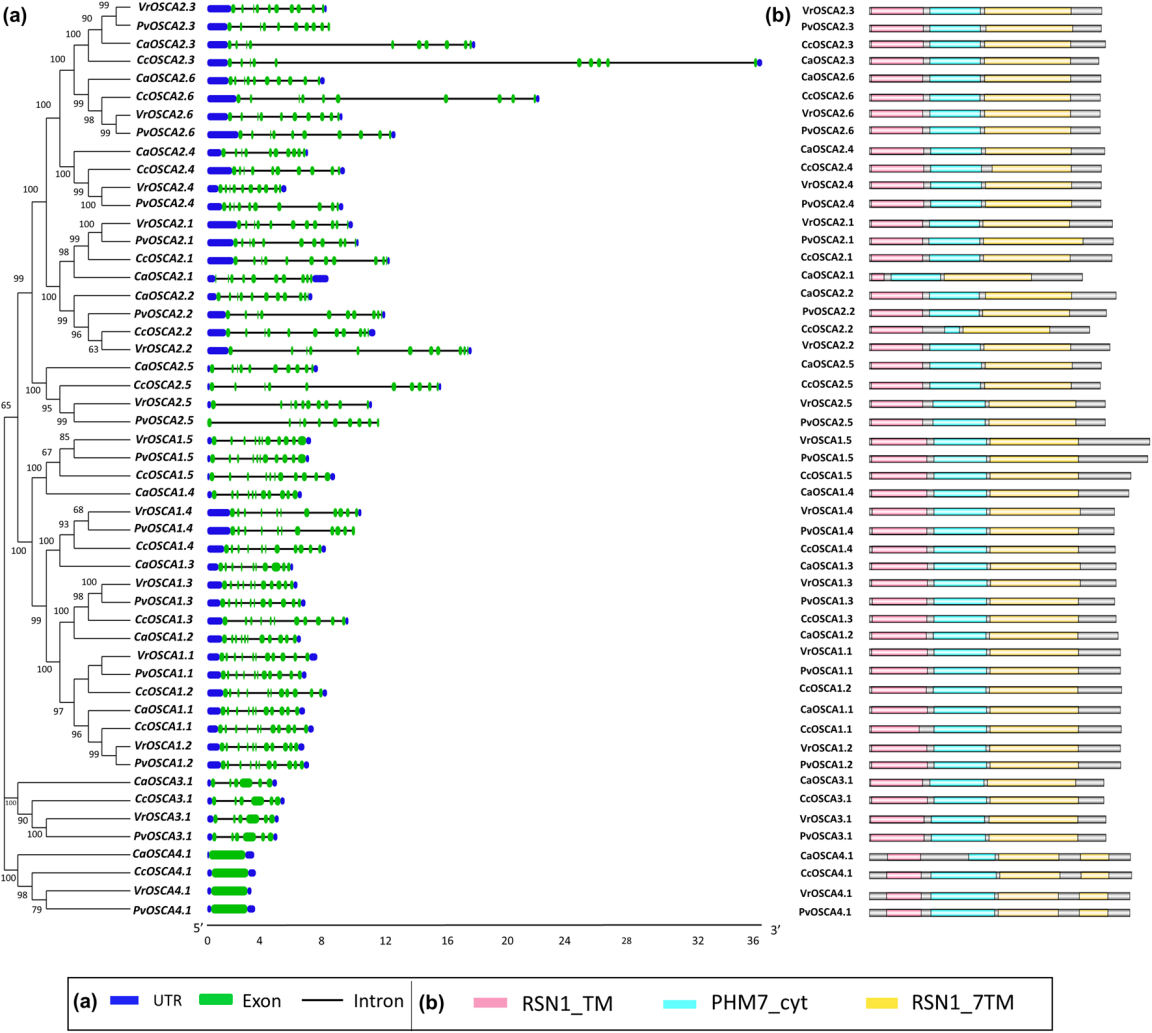
(**a**) Structural features of *OSCA* genes in legumes showing exon–intron organization in 51 *OSCA* genes of four legumes, and gene names are mentioned at the left. Coding sequences (CDS) and untranslated regions (UTR) were indicated by green and blue boxes respectively, and grey lines indicated introns. The scale at the bottom represents gene length in kb; (**b**) Domain organization of the OSCA protein shows three essential domains of OSCA.

Full-length cDNA sequences and their corresponding genomic DNA sequences of all OSCAs were comprehensively analysed to determine the number and position of the exon and introns. The *OSCA4.1* gene in each legume plant species lacks any introns, displaying intron-poor structures. All four *OSCA3.1* genes have five introns and six exons in each plant, showing highly conserved structures. OSCAs present in clades I and II (*CaOSCA1.1–1.4, CaOSCA2.1–2.6, CcOSCA1.1–2.6, VrOSCA1.1–2.6, PvOSCA1.1–2.6*), the number of exons varies from 10–11 (Fig. 2a). Clade I consists of 11 exons, and clade II comprises 10 exons, but this trend has a few exceptions. In *C. arietinum, CaOSCA1.3* comprises 10 exons, indicating an exon loss. Exon gain events are noticeable in six members of group II (*CaOSCA2.2*, *CcOSCA2.1* and *-2.2, VrOsCA2.1* and *-2.2 and PvOSCA2.1*), where each of them consists of 11 exons. The consistency between the number and structure of the exon and introns within the same clades is evident, further implying the structural and functional resemblance between the OSCA proteins in each clade. Similar patterns of exon–intron distribution have been observed in the OSCA family in other plants like rice and cotton^6,14^.

### Three-dimensional structural modeling, domain analysis, and subcellular localization of OSCA proteins

Structural features of the OSCA protein in four legumes reveal that these proteins contain certain distinct regions, including Disordered (18%), Alpha helix (64%), Beta strand (3%), and TM helix (34%). Three-dimensional structure analysis of CaOSCA, CcOSCA, VrOSCA, and PvOSCA proteins reveals that OSCA has a dimeric structure with each monomer consisting of 11 distinct helices, similar to previously characterised proteins in *Arabidopsis* and rice. Two monomers form a V-shaped groove, giving rise to a dimer cavity. This cavity is eight to 20 Å in diameter, and its surface is observed to be hydrophobic. The inner part of the cavity may react with head groups of certain lipids, though the exact function of the dimer cavity needs further investigation^15^ (Supplementary Fig. 1).

The presence of domains strongly influences the structure and functional roles of proteins. In all the OSCA proteins of four aforementioned legume species, three crucial domains viz. RSN1_TM (Calcium permeable stress-gated cation channel 1, PF13967), PHM7_cyt (Cytosolic domain of 10TM putative phosphate region, PF14703), and RSN1_7TM (calcium-dependent channel, 7TM putative phosphate region, PF02714) are present. The SMART tool detected the domain present at N-terminal to be RSN1_TM. Pfam investigation reveals that the RSN1_TM family represents the first three transmembrane regions of 11-TM proteins found to be involved in vesicle transport^7,16^. Similarly, the multi-TM region at the C-terminal end, i.e., RSN1_7TM, is the seven transmembrane domain region, a putative phosphate transporter. The PHM7_cyt domain is located between RSN1_TM and RSN1_7TM. The C-terminal RSN1_7TM is predicted to be the DUF221 domain. Thorough investigation and previous studies ascertain that the DUF-221, or Domain of Unknown function containing hypothetical TM- proteins, is an essential part of any OSCA protein. It consists of an aligned region with approximately 500 amino acid residues^7,17^. All 51 OSCA proteins exhibit a mostly conserved pattern with respect to the presence of essential domains. CaOSCA2.1 was visualized to possess a comparatively smaller RSN1_TM domain. Interestingly, all clade IV members have two DUF221 domains (RSN1_7TM) (Fig. 2b).

The OSCA proteins in each of the four plant species were detected to be present in the plasma membrane of the cell (Supplementary Fig. 2). One of the most distinct characteristic features of the OSCA proteins is the presence of transmembrane helices. CaOSCA proteins are composed of 8–11 transmembrane helices, CcOSCA has 6–10, and both VrOSCA and PvOSCA proteins consist of 7–11 transmembrane helices. The presence of the transmembrane helices further strengthens the predicted subcellular localization of OSCA proteins (Supplementary Fig. 3).

### Motif structure analysis of OSCA protein

Detailed analysis of motif structure information was mined, and 15 unique motif patterns were detected in the 51 OSCA proteins in the four legumes. No motifs were detected below the e-value of 1.1e-1113. Consistent and similar motif anatomy within each clade in each species was predicted, suggesting the existence of clade-specific motifs. In clade I, motifs 3, 7, 4, 13, 6, 12, 2, 9, 5, 1, and 8 are observed in all members. Most members of clade II contain motifs 3, 7, 4, 13, 6, 12, 2, 9, 5, 15, 10, and 11. Motif patterns in clade III are highly consistent, each member consists of motifs 3, 4, 13, 12, 2, 9, 5, 15, and 10. Clade IV (CaOSCA4.1, CcOSCA4.1, VrOSCA4.1, and PvOSCA4.1) proteins reveal exceptional motif structure and composition while maintaining an unfluctuating pattern. They are composed of three motifs only- viz motifs 14, 2, and 13. Motif 14 is unique to clade IV.

These observations suggest that motif 13 is present in all the 51 OSCA proteins studied; hence it must be a part of an essential domain. It is detected as a part of the RSN1_TM domain. Additionally, the RSN1_7TM domain is partially comprised of motif 1 and motif 9. Motif 6 is conserved through the major number of proteins and is a component of the PHM7_cyt domain. Motif 1 is present exclusively in most members of Clade I except for CcOSCA1.1, PvOSCA1.4, and -1.5. Motif 8 is discovered to be unique to all the members of clade 1. Motif 15 is consistently present in clades II and III but absent in all members of clade IV. It is rarely observed in Clade I, with only CaOSCA1.1, CcOSCA1.1 and -1.2, VrOSCA1.1 and -1.2, and PvOSCA 1.1, -1.2, -1.4, and -1.5, demonstrating functional resemblance.

Furthermore, motif patterns in clades I and II are closely similar. CaOSCA2.5, CcOSCA2.5, VrOSCA2.5, and PvOSCA2.5 all lack motifs 7, 6, and 12, which are otherwise present in most members of clade II. Similarly, CaOSCA2.2, CcOSCA2.2, VrOSCA2.2, and PvOSCA2.2 all lack motifs 12. This indicates their functional similarity. This suggests that clade II may have the widest variety of functions. Clades III and IV exhibit recurring motif patterns among themselves, pointing to their distinct roles and evolutionary conserved.

Interestingly, CaOSCA2.1 has a particularly irregular motif pattern, only consisting of motifs 13, 3, 2, 9, 5, 15, 10, and 11. This may support its 3D structure, which is different from all of the other 50 proteins in the study due to the partial presence of helix 2. Only CcOSCA1.1 in *C. cajan* contains motif 10 among the CcOSCA proteins. In *P. vulgaris,* two PvOSCA proteins (PvOSCA1.4 and -1.5) possess motif 10, usually absent in clade I (Supplementary Fig. 4).

### Chromosomal location and gene duplication analysis

The chromosomal location of the *CaOSCA, CcOSCA, VrOSCA,* and *PvOSCA* genes were explored to analyse how they are located within the genome. A total of 48 genes were mapped in accordance with their position in the chromosome (Supplementary Fig. 5). Three genes viz *CaOSCA2.5, CcOSCA3.1,* and *VrOSCA2.4* are present in unknown scaffolds hence they could not be mapped. In *C. arietinum*, *CaOSCA1.2, -2.4,* and *-2.6* is present on chromosome two. Chromosome three (*CaOSCA2.3, -1.3*) and chromosome 7 (*CaOSCA1.1 and -4.1*) contain two genes each. Chromosome one (*CaOSCA3.1*), chromosome four (*CaOSCA2.1*), chromosome five (*CaOSCA2.2*), and chromosome eight (*CaOSCA1.4*) contain one gene each.

In the case of *C. cajan*, *CcOSCA* genes are only present in chromosomes three, seven, nine, 10, and 11. Chromosome three contains the highest number of genes (*CcOSCA1.4, -1.2, -1.3, and -2.2*), followed by chromosome seven which contains three genes (*CcOSCA4.1, -2.4, and -2.3*). Chromosome nine (*CcOSCA2.1 and -2.6*) and chromosome 11 (*CcOSCA1.1 and -1.5*) contain two genes each, whereas chromosome 10 (*CcOSCA2.5*) contains one gene.

In *V. radiata,* the *VrOSCA* genes are present in chromosomes one, three, four, five, six, seven, 10, and 11. Here, chromosome five (*VrOSCA2.5* and *-2.2*), chromosome six (*VrOSCA1.3* and *-2.1*), chromosome seven (*VrOSCA4.1* and *-1.2*), and chromosome ten (*VrOSCA3.1* and *-1.1*) contains two genes each. Chromosome one (*VrOSCA2.6*), chromosome three (*VrOSCA1.4*), chromosome four (*VrOSCA2.3*), and chromosome 11 (*VrOSCA1.5*) contains one gene each.

In the case of *P. vulgaris*, the *PvOSCA* genes are distributed on all the chromosomes from one to nine, except for chromosome seven. Chromosome one (*PvOSCA1.4* and *-2.1*), chromosome two (*PvOSCA1.5* and *-2.4*), chromosome three (*PvOSCA4.1* and *-1.2*), chromosome 6 (*PvOSCA3.1* and *-1.1*) and chromosome 8 (*PvOSCA1.3* and *-2.3*) contains two genes each. Chromosome four (*PvOSCA2.6*), chromosome five (*PvOSCA2.5*), and chromosome nine (*PvOSCA2.2*) contain one gene each.

### Gene duplication events in the OSCA gene family

**Figure 3:**
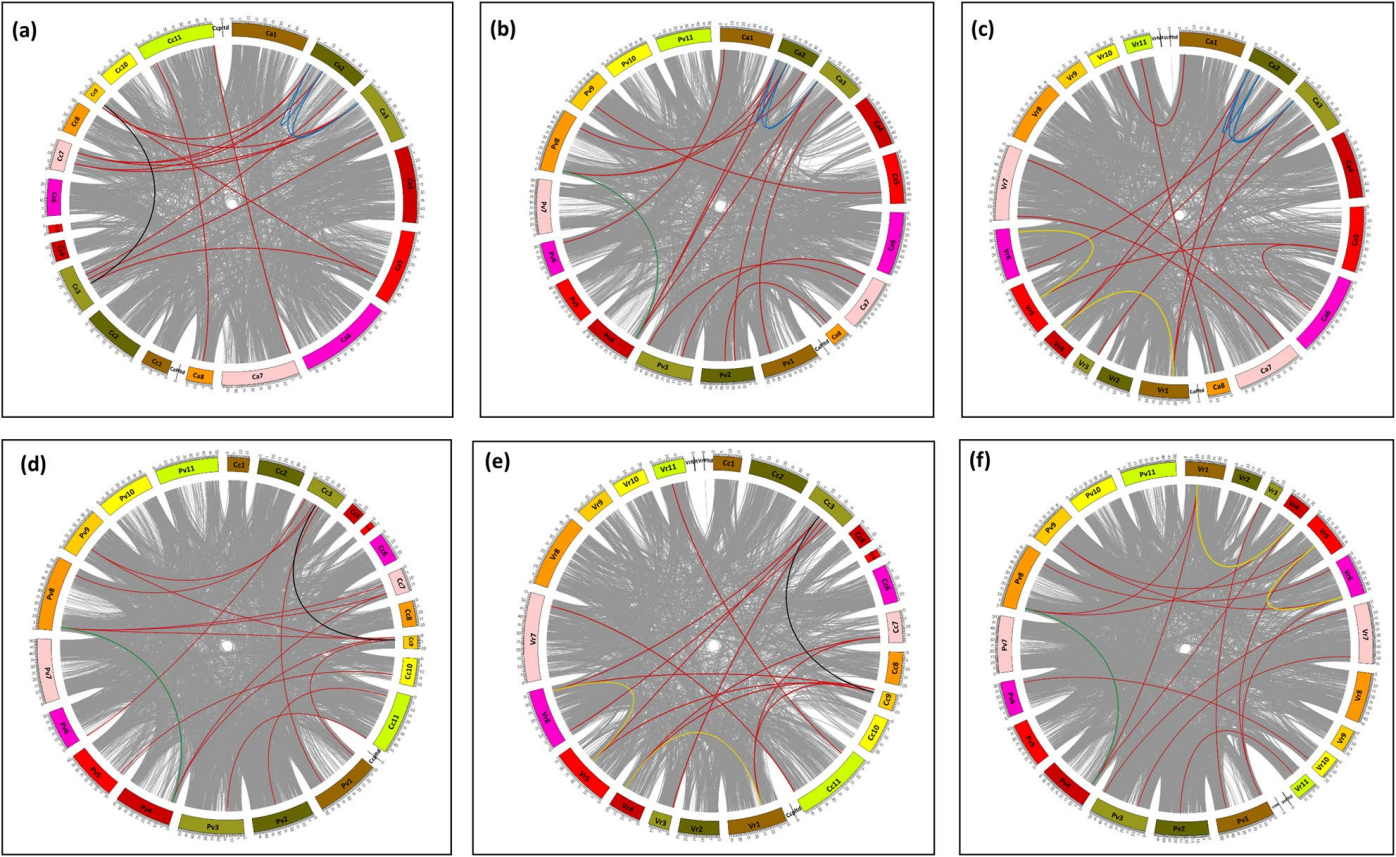
Segmental duplicated gene pairs in OSCAs between, (**a**) *C. arietinum* and *C. cajan,* (**b**) *C. arietinum* and *P. vulgaris,* (**c**) *C. arietinum* and *V. radiata,* (**d**) *C. cajan* and *P. vulgaris,* (**e**) *C. cajan* and *V. radiata,* (**f**) *P. vulgaris* and *V. radiata.* The red lines denote the interspecific gene pairs. The intraspecific duplicated gene pairs are denoted by blue in *C. arietinum,* black in *C. cajan,* yellow in *V. radiata* and green in *P. vulgaris*. The size of each chromosome (Mb) is resembled by the size of the corresponding arc.

Evidence of gene duplication are noticeably present in the OSCA gene family in *C. arietinum, C cajan, V. radiata, and P. vulgaris*. A thorough investigation of the genomes of the four plant species reveals that all four genomes have undergone segmental duplication events giving rise to both interspecific and interspecific gene pairs. A total of 78 pairs of interspecifically duplicated genes were identified, which include 13 between *C. arietinum* and *C. cajan*, 13 between *C. arietinum* and *P. vulgaris*, 12 between *C. arietinum* and *V. radiata*, *C. cajan,* and *P. vulgaris* showing 14, *V. radiata* and *C. cajan* showing 12, and 14 between *P. vulgaris* and *V. radiata*. Moreover, among the four plant species, *C. arietinum* contains three (*CaOSCA2.4–2.6*, *CaOSCA2.4–2.3,* and *CaOSCA2.3–2.6)*, *C. cajan* contains one (*CcOSCA2.2–2.1*), *V. radiata* contains two (*VrOSCA2.1–2.2* and *VrOSCA2.3–2.6*), and *P. vulgaris* contains one (*PvOSCA2.3–2.6*) intraspecific gene duplication pairs (Fig. 3).

The three types of gene duplication observed in the *OSCA* gene family are tandem duplication, whole genome (WGD) or segmental duplication, and dispersed duplication, i.e., those which cannot be categorized as tandem, proximal, or whole genome duplications. In Clade I, all the genes show either tandem or dispersed gene duplication. *CaOSCA1.1, -1.2,* and *1.4* in *C. arietinum, CcOSCA1.1* in *C. cajan, VrOSCA1.1, -1.2* in *V. radiata,* and *PvOSCA1.4* in *P. vulgaris* originated as a result of tandem duplication. *CaOSCA1.3, VrOSCA1.3, CcOSCA1.3,* and *PvOSCA1.3* are a product of the dispersed type of duplication. *CcOSCA1.2, -1.4, -1.5, VrOSCA1.3, -1.4 -1.5,* and *PvOSCA1.1, -1.2,* and *-1.5* originated due to dispersed duplication. In Clade II, most genes have originated by tandem and whole genome duplication, except *PvOSCA2.4* and *-2.5*, which show dispersed duplication. Interestingly, all the genes in four legumes involved in intraspecific gene duplication (*CaOSCA2.3, -2.4*, *-2.6, CcOSCA2.1, -2.2*, *VrOSCA2.1, -2.2, -2.3, -2.6*, and *PvOSCA2.3, -2.6*) are found to have originated as a result of whole genome duplication events. Apart from that, *CaOSCA2.1, -2.5*, *CcOSCA2.4, -2.5, -2.6, VrOSCA2.4, -2.5*, and *PvOSCA2.1* emerged as a result of tandem duplication. Intriguingly, all Clade III and IV members emerged due to dispersed duplication events.

**Figure 4:**
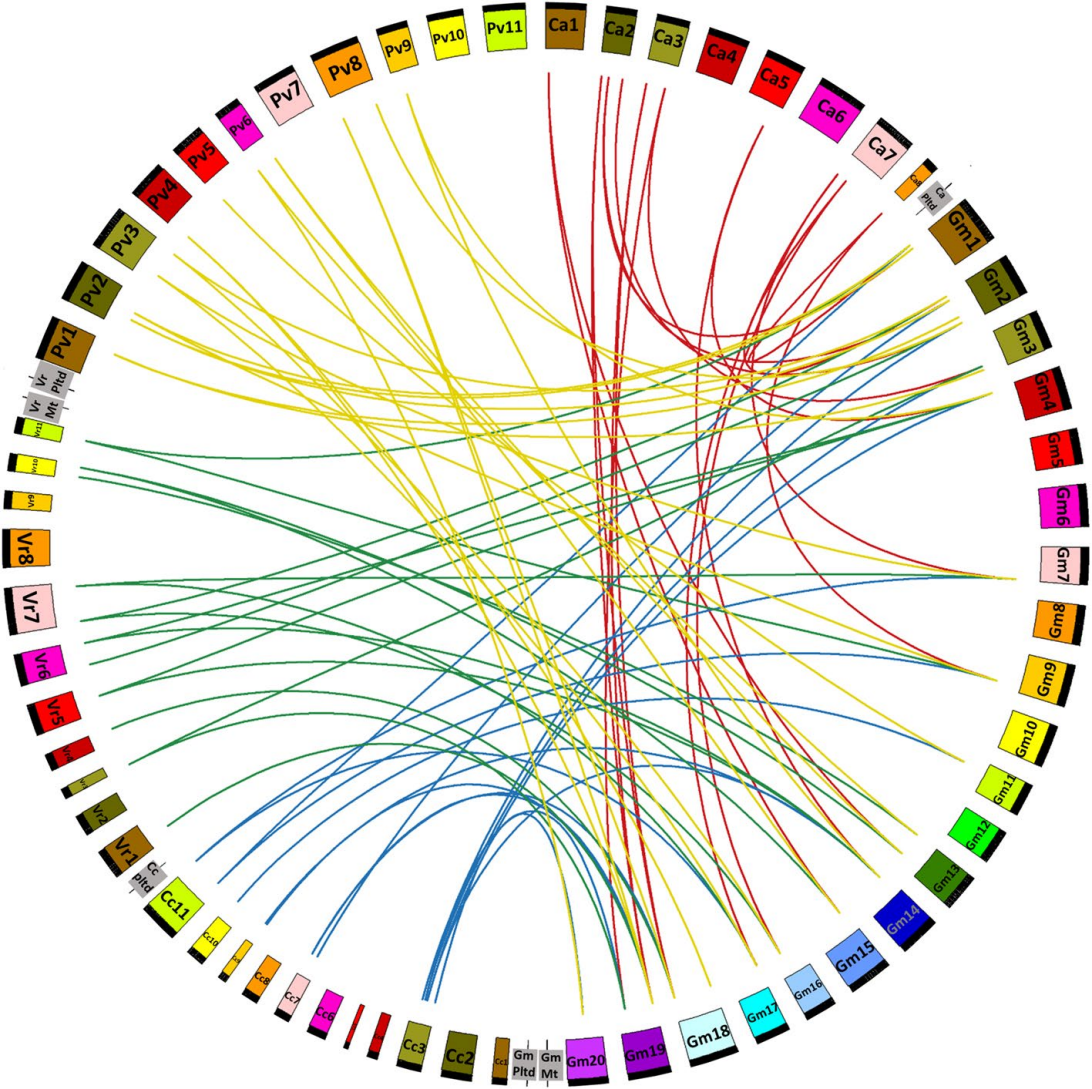
Segmentally duplicated OSCA gene pairs between *C. arietinum* and *G. max* (red), *C. cajan* and *G. max* (blue)*, V. radiata* and *G. max* (green)*, P. vulgaris* and *G. max* (yellow).

The evolutionary process of the OSCA genes is further emphasized by collinearity analysis between the four target legumes of our study and *G. max* (soybean). A total of 76 gene pairs were detected between the five legumes, including 19 pairs between *C. arietinum* and *G. max,*16 pairs between *C. cajan* and *G. max*, 19 pairs between *V. radiata and G. max,* and 22 pairs between *P. vulgaris and G. max* (Fig. 4 and Supplementary Table 2c)*.* Most *GmOSCA* genes reveal instances of collinear relationship with the *CaOSCAs, CcOSCAs, VrOSCAs* and *PvOSCAs*. *GmOSCA1.3* shows no collinearity with the chickpea and pigeon pea genome but shares segmentally duplicated regions with mung bean and common bean. Additionally, *PvOSCA2.1* and *CaOSCA2.1* do not share any collinearity with the *GmOSCA*s. The results indicate that most OSCA genes in the legumes have evolved due to gene duplication events, but a few might be present earlier.

To gauge the pressure of evolutionary selection on the OSCA genes, Ka (nonsynonymous) and Ks (synonymous) values were calculated. Ka/Ks ratio, or the substitution rate ratio, was calculated for each of the 78 interspecific and the seven intraspecific collinear gene pairs. All 78 interspecific duplicated gene pairs and seven pairs of paralogous genes exhibited a ka/ks ratio of less than one in each case (Supplementary Table 2). Therefore, all the segmentally duplicated OSCA genes have been subjected to purifying selection pressure^7,18^.

### Analysis of cis-regulatory elements

A total of 16 consensus cis-regulatory elements were identified in the 2 kb upstream region of the OSCA genes in *C. arietinum*, *C. cajan*, *V. radiata,* and *P. vulgaris* (Supplementary Fig. 6 and Supplementary Table 3). They include six stress-responsive factors namely G-box (pathogen inducible element/positive regulator of senescence), LTR (low temperature-responsive element), MBS (MYB binding site in drought-inducibility), WUN-motif (wound-responsive element), STRE (stress-responsive element), and TC-rich repeats (defence and stress response), six hormone-responsive elements including ABRE (Abscisic acid responsive element), CGTCA/TGACG-motif (methyl jasmonate responsive element), TGA-element (auxin-responsive element), TCA-element (salicylic-acid responsive element) and ERE (ethylene-responsive element), four growth and development factors such as O2-site (anaerobic induction), GCN4_motif (endosperm expression), ARE (anaerobic induction) and Box-4 (element related to light-responsiveness)^19,20^.

All four legumes contain abundant ABRE, G-box, ARE, and Box 4. In *C. arietinum,* STRE is rarely present but is consistently present in *C. cajan, V. radiata,* and *P. vulgaris.* Two plant growth-related elements ARE and Box4, are found abundantly in the promoter of most *OSCA* genes.

### Gene ontology (GO) analysis

We conducted a GO analysis of the 51 OSCA proteins. A total of 21 GO terms were identified including six biological processes, 12 molecular functions, and three cellular components. With 51 proteins displaying this activity, cation transmembrane transport is predicted to be the most prevalent biological process undertaken by the *OSCA* gene family. CaOSCA2.2 is detected to have RNA biosynthetic properties, which is absent in any other *OSCA* genes in all the 51 genes studied. Interestingly, members of group IV in each legume are observed to have the most varied functions. They were predicted to execute roles like protein targeting to the vacuole, hydrolysis of RNA phosphodiester bond, endonucleolytic processes, and proteolysis, along with cation transmembrane transport activity, indicating a very diverse range of functions. CcOSCA2.1, VrOSCA2.1, and PvOSCA2.1 are detected to regulate other biosynthetic processes. The most distinct molecular function of the OSCAs was detected to be calcium-activated cation channel activity, as displayed by all 51 OSCA proteins. Additionally, they are also predicted to perform various other molecular functions. RNA–DNA hybrid ribonuclease activity, aspartic-type endopeptidase activity, and nucleic acid binding activity are common and unique to all the group IV members in all four legumes. Only CaOSCA1.1 and PvOSCA1.1 were predicted to have mechanosensitive ion channel activity and identical protein binding activity. However, pyridoxal phosphate binding activity is prominent in CcOSCA2.1, VrOSCA2.1, and PvOSCA2.1 but is absent in CaOSCA2.1. Intriguingly, enzymatic activities are also detected to be performed by the OSCAs. Similar results are observed in recent studies where OSCAs are discovered to be associated with NAD(P)-dependent dehydrogenase in *Zea mays*^21^*.* As for cellular composition, all 51 OSCA proteins are predicted to be an integral component of the plasma membrane, whereas CaOSCA2.2 might also be a part of the DNA-directed RNA polymerase complex.

In *C. arietinum*, CaOSCA1.1, -1.3, -2.4, and **-**3.1 are predicted to exhibit beta-maltose 4-alpha-glucanotransferase activity and 4-alpha-glucanotransferase activity. Other functions of the CaOSCAs include oxidoreductase activity exhibited by CaOSCA1.1, -1.2, -2.2, -2.3, -2.4, -2.6, and -4.1, hydrolase activity displayed by CaOSCA2.2. Unlike the other three legumes, pyridoxal phosphate binding activity is not observed in chickpea. DNA-directed 5'-3' RNA polymerase activity is unique only to CaOSCA2.2 and absent in all other OSCA genes in the study.

In *C. cajan,* beta-maltose 4-alpha-glucanotransferase activity and 4-alpha-glucanotransferase activity are observed in CcOSCA1.1, -1.2, -1.4, -2.4, and -3.1*,* hydrolase activity is predicted in CcOSCA2.1, and -2.2*.* Oxidoreductase activity is exhibited by CcOSCA1.1, -1.2, -1.3, -2.2, -2.3, -2.4, -2.6, and **-**4.1*.* Mechanosensitive channels and identical protein binding activity are absent in pigeon pea.

In *V. radiata*, VrOSCA1.1. -1.2, -1.4, -2.4, and -3.1 displays beta-maltose 4-alpha-glucanotransferase activity and 4-alpha-glucanotransferase activity. Similar to pigeon pea, oxidoreductase activity is exhibited by VrOSCA1.1, -1.2, -1.3, -2.2, -2.3, -2.4, -2.6, and **-**4.1 in mungbean. VrOSCA2.1 and VrOSCA2.2 perform hydrolase activities.

In *P. vulgaris,* PvOSCA1.1. -1.2, 1.4, 2.4, 3.1 exhibits beta-maltose 4-alpha-glucanotransferase activity and 4-alpha-glucanotransferase activity, and oxidoreductase activity is demonstrated by PvOSCA1.1, -1.2, -1.3, -2.2, -2.3, -2.4, -2.6 and **-**4.1. No hydrolase activity is observed in *PvOSCA* genes (Supplementary Fig. 7 and Supplementary Table 4).

### Expression profile of *OSCA* genes in different developmental stages

**Figure 5:**
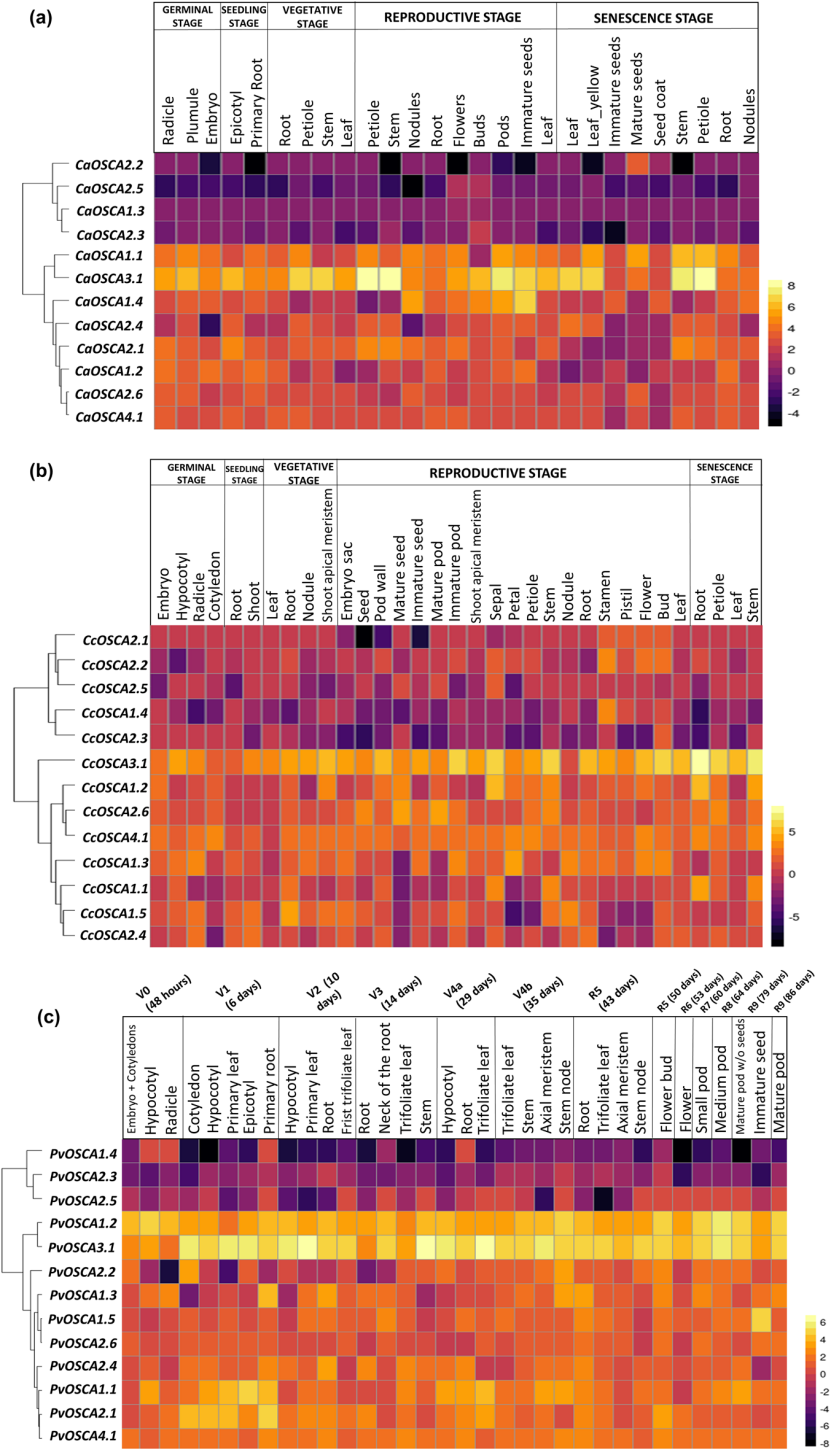
Gene expression analysis at different developmental stages. The genes are named on the left and different tissues/developmental stages are labelled at the top. The scale bar represents the normalised log2 FPKM values; (**a**) The heatmap represents the expression pattern of *CaOSCA* genes of *Cicer arietinum* at different developmental stages, such as germination, seedling, vegetative, reproduction and senescence; (**b**) Expression profiles of different *CcOSCA* genes in different developmental stages of *Cajanus cajan*. The heatmap represents the expression pattern of *CcOSCA* genes at developmental stages such as germination, seedling, vegetative, reproduction and senescence; (**c**) Expression profiles of different *PvOSCA* genes in different developmental stages of *Phaseolus vulgaris*. The heatmap represents the expression pattern of *PvOSCA* genes at different time periods in different tissues a different age of plants.

Expression profile analysis was conducted in *C. arietinum*, *C. cajan,* and *P. vulgaris* about their developmental stages and response to abiotic and biotic stress conditions. In *V. radiata,* expression analysis was studied in stress conditions but not in the case of developmental stages due to insufficient data. In *C. arietinum*, 27 tissues were taken into account for expression analysis studies at the following developmental stage- germinal stage (radicle, plumule, embryo), seedling stage (epicotyl, primary root), vegetative stage (petiole, stem, leaf, root), reproductive stage (leaf, petiole, stem, nodules, root, flower, bud, pod, immature seed), and senescence stage (leaf, leaf-yellow, immature seed, mature seed, seed coat, stem, petiole, root, nodule) from BioProject PRJNA413872, which studied the gene expression analysis of chickpea in different developmental stages. Most *CaOSCA* genes are considerably upregulated, with *CaOSCA3.1* showing the highest level of upregulation. However, *CaOSCA2.6* and *CaOSCA4.1* shows no significant change all along in the tissues (Fig. 5a and Supplementary Table 5a).

In *C. cajan*, RNA-Seq data of 33 tissues from BioProjects PRJNA344973 and PRJNA354681 were considered for expression analysis studies at different developmental stages, namely germinal stage (embryo, hypocotyl, radicle, and cotyledon), seedling (root and shoot), vegetative stage (leaf, root, nodule, and shoot apical meristem), reproductive stage (embryo sac, seed, pod wall, mature seed, immature seed, mature pod, immature pod, shoot apical meristem, sepal, petal, petiole, stem, nodule, root, pistil, stamen, flower, bud, and leaf), and senescence stage (root, petiole, leaf, and stem). Most genes are commonly upregulated, *CcOSCA1.2, -2.3, -3.1,* and *4.1* genes show consistently elevated expression in most tissues (Fig. 5b and Supplementary Table 5b). Experiments in both the BioProjects used in this study were carried out in *Cajanus* genotype Asha. BioProject PRJNA344973 elucidated the gene expression in the embryo sac, seed, and pod wall at, while BioProject PRJNA354681 developed a gene expression atlas representing different developmental stages.

In *P. vulgaris*, expression analysis of *OSCA* genes was conducted at different stages of development has been studied in 34 tissues at the following time intervals given as follows- embryo + cotyledons, hypocotyl, and radicle at V0(48 h); cotyledons, hypocotyl, primary leaf, epicotyl and primary root at V1(6 days); hypocotyl, primary leaf, root and first trifoliate leaf at V2(10 days); root, neck of the root, trifoliate leaf, stem at V3(14 days); hypocotyl, root and trifoliate leaf at V4a(29 days); trifoliate leaf, stem, axial meristem, and stem node at V4b(35 days); root, trifoliate leaf, axial meristem, and stem node at R5(43 days); flower bud at R5(50 days); flower at R6(53 days); small pod at R7(60 days); medium pod at R8(64 days); mature pod without seeds and immature seeds at R9(79 days) and mature pod at R9(86 days). *PvOSCA-1.1, 1.2, 2.1, 2.4, 3.1,* and *4.1* genes are upregulated throughout each tissue. *PvOSCA1.4* gene shows diminished expression in all the tissues. The NCBI SRA data was extracted from BioProject PRJNA221782, which analyzed the gene expression in a Mesoamerican *P. vulgaris* variant BAT93 at different developmental intervals (Fig. 5c and Supplementary Table 5c).

### Expression profile of *CaOSCA* under abiotic stress

**Figure 6:**
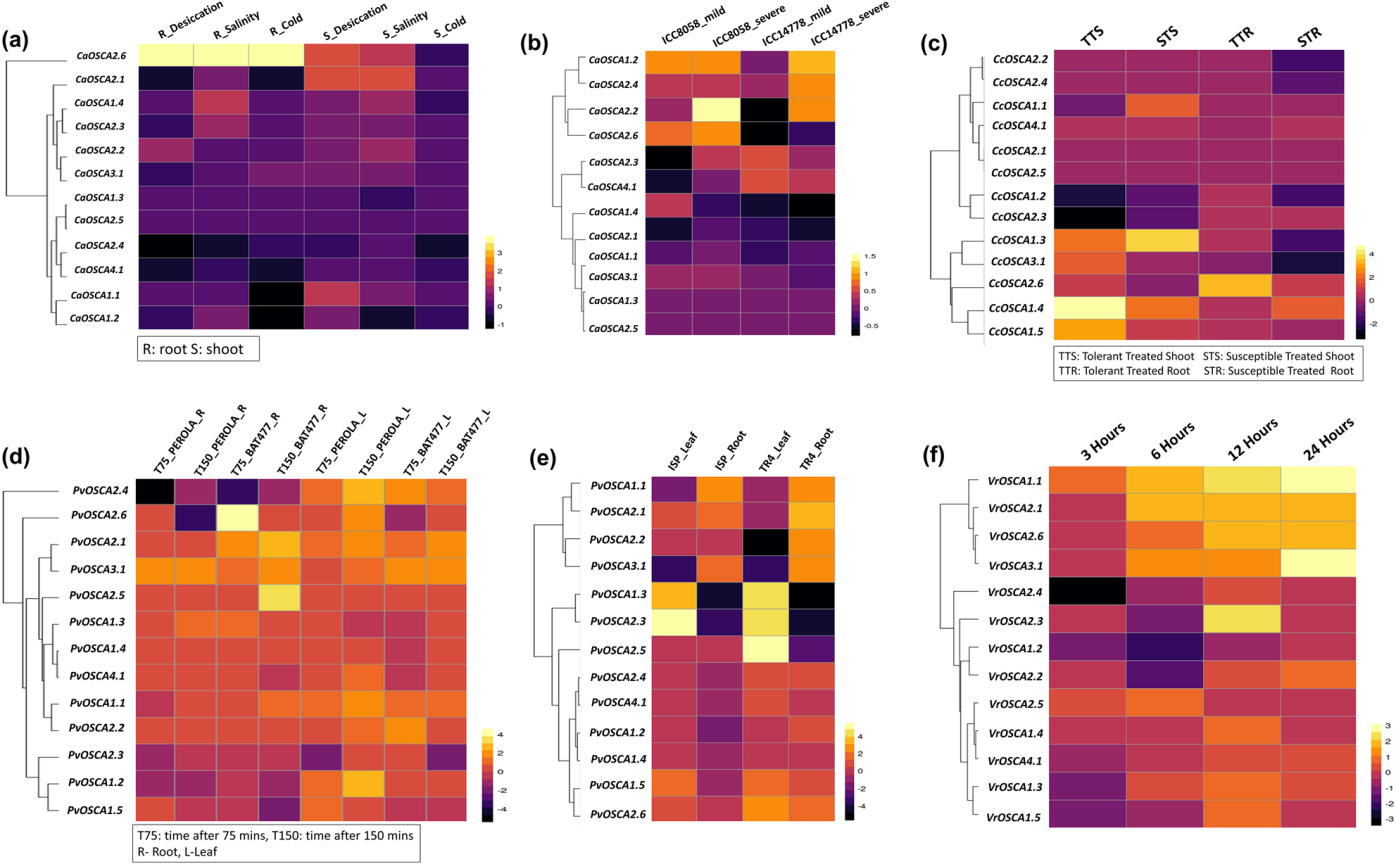
Expression profiles of *OSCA* genes in different abiotic stresses. The genes are named on the left, and condition/tissue is labelled at the top. The scale bar represents the log_2 Fold Change based on FPKM values; (**a**) Expression pattern of *CaOSCA* genes in three abiotic stress -cold, desiccation and salinity in the root (R) and shoot (S); (**b**) Expression pattern of *CaOSCA* genes in mild and severe drought stress; (**c**) Expression profiles of *CcOSCA* genes in salinity stress in ICP 7-salt-tolerant genotype (indicated by TTS-Tolerant treated shoot and TTR- Tolerant treated root), ICP 1071-Salt susceptible genotype (indicated by STS-Susceptible treated shoot, STR-Susceptible treated root); (**d**) Expression profiles *PvOSCA* genes in drought stress in two genotypes at two different time points; (**e**) Expression profiles of *PvOSCA* genes in salinity stress in the root (R) and leaf (L) in two genotypes; (**f**) Expression profiles of *VrOSCA* genes in desiccation stress at four different time points.

In *C. arietinum*, abiotic stress regulation was studied in BioProject PRJNA232700, which investigated the transcriptomic dynamics under desiccation, salinity, and cold stress in the root and shoot of cultivar ICC4958. In *C. arietinum*, abiotic stress regulation was studied in cultivar ICC4958 in root and shoot against desiccation, salinity, and cold stress using RNA-Seq data from BioProject PRJNA232700. *CaOSCA2.6* is greatly upregulated in the case of all three stress conditions in both roots and shoot except for displaying downregulated expression in cold stress in shoot tissue. The caOSCA1.1 gene is considerably upregulated in desiccation stress in the shoot. *CaOSCA2.3* is upregulated in salt stress in root tissue (Fig. 6a and Supplementary Table 6a.1).

BioProject PRJNA436616 performed transcriptome sequencing of drought-susceptible and drought-resistant chickpea cultivars under drought conditions. SRA data from it was extracted to investigate the regulatory roles of *CaOSCA* under mild and severe drought conditions in cultivars ICC 8058 and ICC 14,778. *CaOSCA2.2* shows upregulated activity under severe drought conditions in cultivar ICC 8058 (Fig. 6b and Supplementary Table 6a.2).

### qRT-PCR expression analysis under abiotic stress (desiccation, salinity, and cold stress)

**Figure 7:**
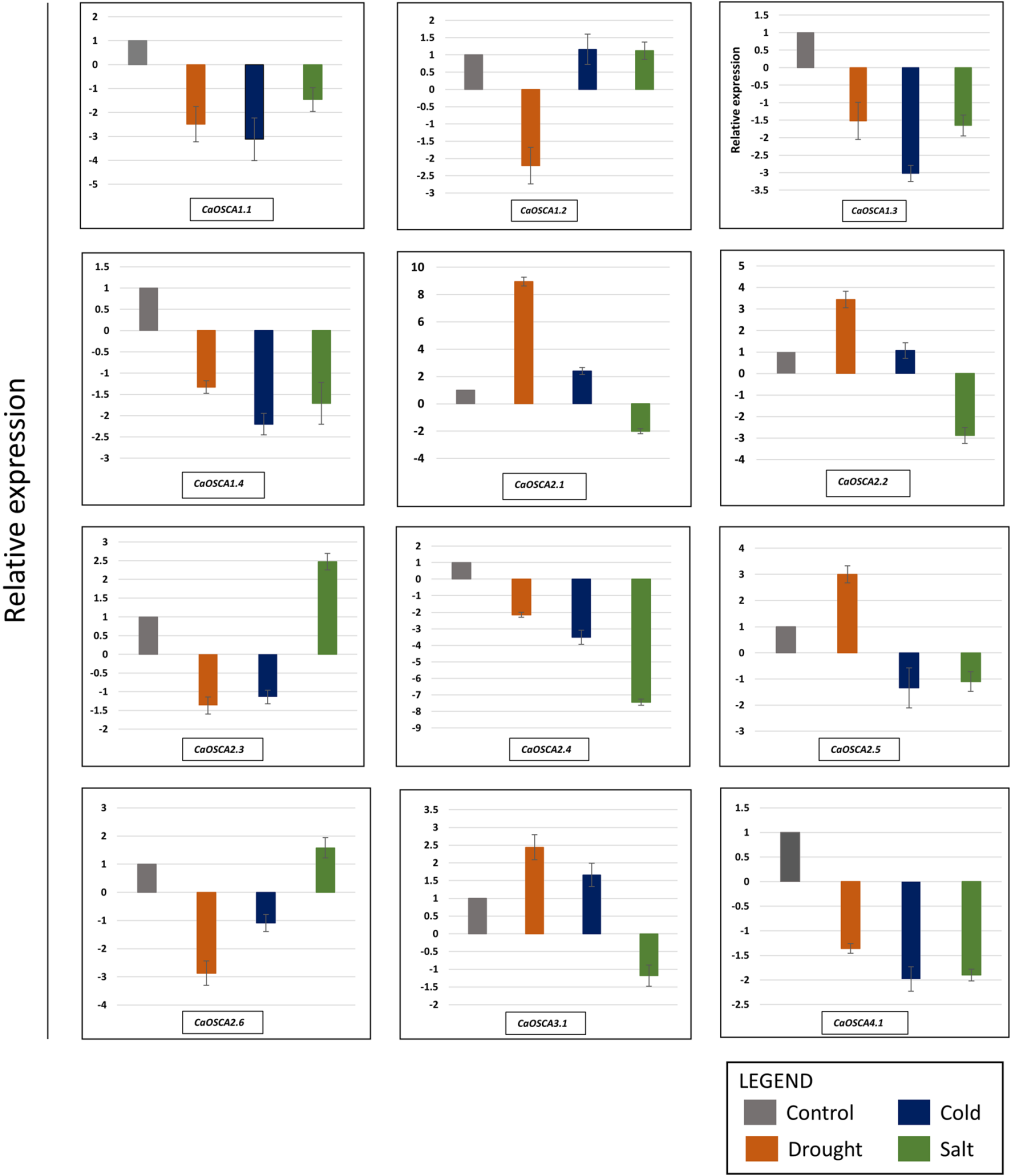
Bar plot showing expression of *OSCA* genes under desiccation (orange), cold (dark blue), and salt (green). Control is shown in grey. Y- axis depicts relative expression. Results are obtained from qRT-PCR data.

To better understand the role of *OSCA* genes in abiotic stress conditions, qRT-PCR analysis was performed for all 12 *CaOSCA* genes under desiccation, cold, and salinity stress. Expression data were obtained from 21-day-old seedlings exposed to the aforementioned stress factors. All the clade I *CaOSCA* genes are downregulated under desiccation stress, but in clade II, the *CaOSCA2.1* gene is highly upregulated, followed by *CaOSCA2.2, -2.5,* and **-***3.1*. Exposure to cold stress results in the upregulation of *CaOSCA1.2, -2.1, -2.2,* and **-***3.1* genes. Three genes viz *CaOSCA1.2, -2.3,* and *-2.6* are upregulated when subjected to salt stress; however, all the other genes are downregulated (Fig. 7).

### Expression profile of *CcOSCA* in abiotic stress

In the case of *C. cajan*, abiotic stress regulation was studied in 4-week-old seedlings of ICP 7(Salt tolerant pigeon pea genotype) and ICP 1071(Salt susceptible pigeon pea genotype) subjected to salinity stress utilizing transcriptomic data from BioProject PRJNA382795 and BioProject PRJNA343064. BioProject PRJNA382795 explores the transcriptome of salt-tolerant (ICP 7) and salt-susceptible (ICP 1071) *C. cajan* genotypes in the root, whereas BioProject PRJNA343064 investigates the same in shoot tissues. Three genes, namely *CcOSCA1.3, -1.4,* and *-1.5,* are upregulated in the shoot of both genotypes. *CcOSCA3.1* shows an elevated expression in the ICP7 shoot. *CcOSCA2*.*6* is upregulated in the root of the tolerant genotype. *OSCA* genes are more commonly downregulated in roots of the salt-susceptible genotype than in salt-tolerant genotype (Fig. 6c and Supplementary Table 6b).

### Expression profile of *PvOSCA* in abiotic stresses (drought and salt stress)

The drought stress regulatory role of the *PvOSCA* genes is investigated in two different genotypes, BAT 477 (drought-tolerant) and Perola (drought-susceptible) using publicly available data from BioProject PRJNA327176, aimed at elucidating the contrasting gene expression of the two above-mentioned Mesoamerican common bean genotypes under drought stress. The study was conducted in leaf and root tissues at two different time points, i.e., expression after 75 min and expression after 150 min of being subjected to stress conditions. Most *PvOSCA* genes are expressed in both genotypes' tissues at two different time frames. *PvOSCA2.4* gene is downregulated in the root of both genotypes after both time intervals but is upregulated in the leaves of both genotypes at both time intervals. *PvOSCA2.6* is upregulated in the first 75 min but is downregulated after 150 min in the root of BAT 477. Contradictorily, the *PvOSCA2.5* gene is moderately expressed after 75 min, but exhibits elevated upregulation at 150 min in BAT 477 root. *PvOSCA2.1* and *PvOSCA3.1* are consistently expressed in both the tissues of the two genotypes in the study at two different time points, with *PvOSCA3.1* showing the highest expression in BAT 477 leaf and *PvOSCA2.1* being highly upregulated in BAT 477 root, after being exposed to 150 min of drought stress. In genotype Perola, *PvOSCA1.1,* and *PvOSCA1.2* are upregulated in the leaf after 150 min of stress (Fig. 6d and Supplementary Table 6c.1).

In *P. vulgaris*, expression analysis was conducted in the leaf and root tissue of two different genotypes, Ispir (tolerant) and TR43477 (susceptible), when subjected to salinity stress using RNA-Seq data from BioProject PRJNA656794, which analyses salinity regulation mechanisms in salt-tolerant (Ispir) and salt-susceptible (TR43477) common bean genotypes. Most *PvOSCA* genes are considerably overexpressed when subjected to salinity stress. Five *PvOSCA* genes (*PvOSCA1.3, -1.5, -2.3,* and *-2.5*) are expressed in the leaf of TR43477. *PvOSCA2.6* is upregulated in both leaf and root tissues of TR43477. Three genes, *PvOSCA1.1* and *-3.1, are* upregulated in the root of Ispir genotype, and *PvOSCA2.1* is upregulated in both tissues of Ispir genotype. Two genes, *PvOSCA1.3 and -2.3,* are commonly upregulated in both genotypes' leaf tissue, but contradictorily, they are downregulated in the roots. Two genes viz *PvOSCA1.1* and *3.1* genes are downregulated in leaf tissue of both the genotypes, but in the case of root, they exhibit substantially upregulated expression. (Fig. 6e and Supplementary Table 6c.2).

### Expression profile of *VrOSCA* in abiotic stress

To explore the possible role of the *VrOSCA* genes in abiotic and biotic stress regulation, expression levels of 13 *VrOSCA* genes were explored. For this, NCBI SRA repository data from BioProject PRJNA327304 were utilized, where seeds of mungbean variety Zhonglu 1 were subjected to desiccation following imbibition for three (SY3H), six (SY6H), 18 (SY18H), and 24 h (SY24H), and then their transcriptional dynamics were compared. Seeds of mungbean variety Zhonglu 1 were subjected to desiccation following imbibition for three (SY3H), six (SY6H), 18 (SY18H), and 24 h (SY24H), and then their transcriptional dynamics were compared. At least four genes viz *VrOSCA1.1, VrOSCA2.1, VrOSCA3.1,* and *VrOSCA2.6* are upregulated in 6, 18, and 24-h imbibed seeds, where the highest degree of upregulation is observed in SY24H seeds. *VrOSCA2.3* was upregulated in SY18H seeds but showed no expression in SY8H and SY24H seeds and is downregulated in SY6H seeds (Fig. 6f and Supplementary Table 6d).

### Expression analysis of *CaOSCA* genes in biotic stress conditions

**Figure 8:**
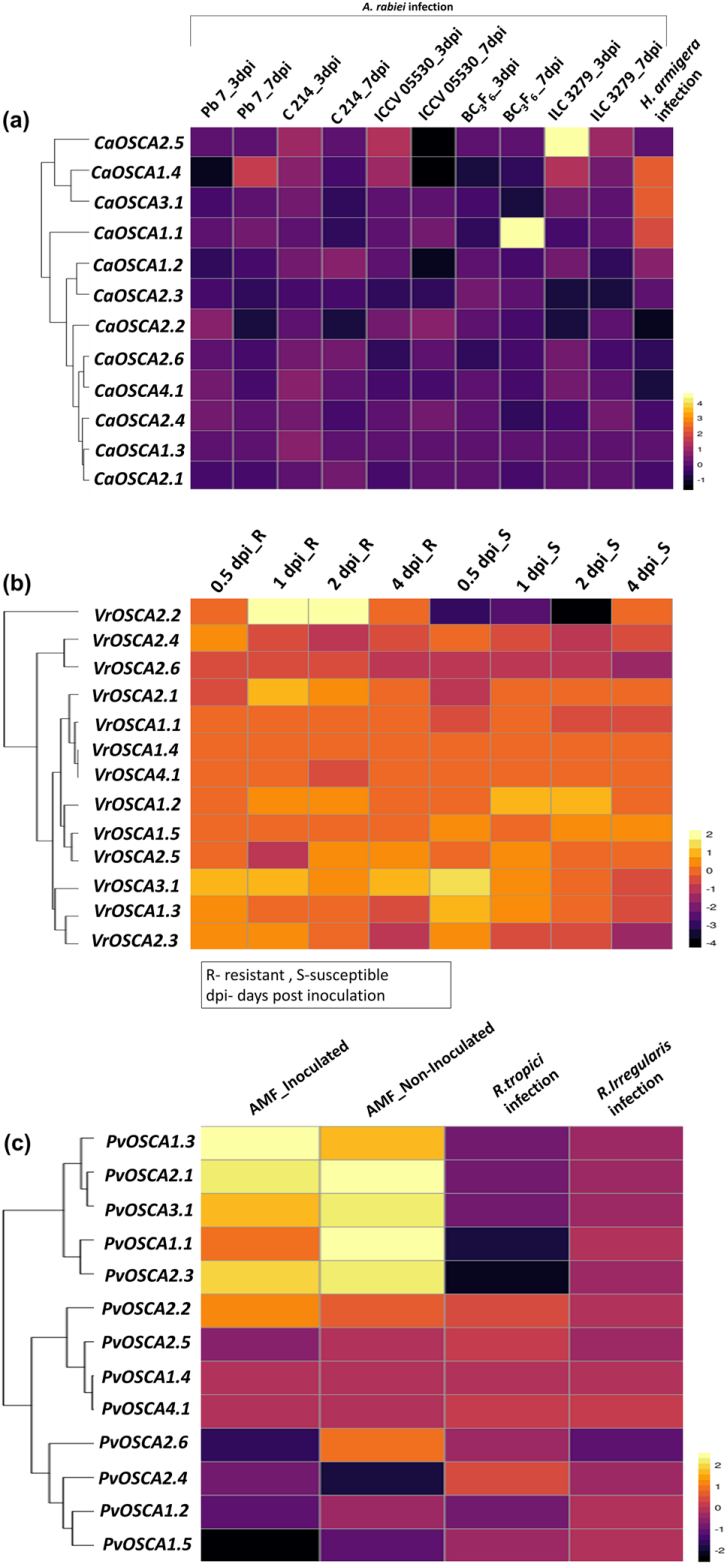
Expression profiles of *OSCA* genes in different biotic stresses. The genes are named on the left, and condition/tissue is labelled at the top. The scale bar represents the log_2 Fold Change based on FPKM values; (**a**) Expression pattern of *CaOSCA* genes under *Ascochyta rabiei* infection in five chickpea cultivars; under *Helicoverpa armigera* infection; (**b**) Expression pattern of *VrOSCA* genes under *Fusarium oxysporium* infection at four different time points. (**c**) Expression pattern of *PvOSCA* genes under the cumulative effect of drought stress and AMF; and *Rhizobium tropici* and *Rhizophagus irregularis* infections.

*CaOSCA* genes are not significantly expressed when exposed to *Ascochyta rabiei* infection. Leaf and stem tissues of two susceptible genotypes, namely Pb 7, C 214, two resistant genotypes-ICCV 05530 and ILC3279, and BC3F6, an *A. rabiei* resistant introgression line, were considered. The expression was studied after three- and seven-days post-inoculation by *A. rabiei* utilizing data from BioProject PRJNA479940, which aimed at revealing the interplay between *A. rabiei* fungi and *C. arietinum*. *CaOSCA1.1* is highly expressed in genotype BC3F6 seven days post-inoculation. *CaOSCA2.5* is highly upregulated in genotype ILC 3279 three days post-inoculation. *CaOSCA1.4* has the most variable expression. It is upregulated in Pb 7 seven days post-inoculation, but C214, ICCV 05530, and ILC3279 show elevated expression three days post-inoculation. Other *CaOSCA* genes are mostly downregulated (Fig. 8a and Supplementary Table 7a.1).

*CaOSCA* gene expression in biotic stress was explored under *Helicoverpa* infection with NCBI SRA data extracted from BioProject PRJNA328302. Leaves from eight-week-old chickpea plants were wounded and exposed to bollworm infection, and then their transcript abundance was quantified in BioProject PRJNA328302. *CaOSCA1.3, -2.3* and *2.5* shows no expression*.* Four genes *CaOSCA1.1, CaOSCA1.2, CaOSCA1.4,* and *CaOSCA3.1* are significantly upregulated (Fig. 8a and Supplementary Table 7a.2).

### Expression analysis of *VrOSCA* genes in biotic stress conditions

The possible role of *VrOSCA* genes in biotic stress is investigated by utilizing the NCBI repository data available in BioProject PRJNA715596, which explored mung bean lines Zheng8-4(resistant) and Zheng8-20(susceptible) subjected to *Fusarium oxysporium* infection and the expression levels of the *VrOSCA* genes are compared at 0, 0.5, 1-, 2-, and 4-days post inoculation(dpi). The *VrOSCA2.2* is upregulated at 1 dpi and 2 dpi but shows no expression after 4 days of inoculation in the resistant line but is mostly downregulated in the susceptible line. *VrOSCA3.1* is mostly expressed in both lines with an upregulated expression in Zheng8-20 at 0.5 dpi but is downregulated after 24 days of exposure to infection. The *VrOSCA* genes are mostly downregulated in the susceptible line Zheng8-20 than in the resistant line Zheng8-4 (Fig. 8b and Supplementary Table 7b).

### Expression profile of *PvOSCA* in cumulative abiotic and biotic stress conditions

Expression of *PvOSCA* genes is studied when the plant is subjected to both abiotic and biotic stress conditions by extracting transcriptomic data from BioProject PRJNA311998, which examined the impact of AMF symbiosis on a drought-tolerant *P.vulgaris* variety-*BAT 477*. The root of genotype *BAT 477* was inoculated with AMF (arbuscular mycorrhizal fungi) and was subjected to drought stress, and their expression were studied after 42 days. More *PvOSCA* genes are expressed in plants non-inoculated with AMF. *PvOSCA1.1, -1.3, -2.1, -2.2, -2.3,* and *-3.1* are expressed in both AMF-inoculated and AMF non-inoculated conditions with varying expression levels (Fig. 8c and Supplementary Table 7c).

### Expression analysis of *PvOSCA* genes in biotic stress conditions

Expression of *PvOSCA* genes has been investigated in case of biotic stress conditions using transcriptomic data from BioProject PRJNA482464, which investigates the effect of increased ROS on the symbiotic relationship between *P. vulgaris- Rhizobium tropici* and *P. vulgaris- Rhizophagus irregularis*. Transgenic root in *P. vulgaris* cv *Negro jamapa* was inoculated with *Rhizobium tropici* and an AMF-*Rhizophagus irregularis,* and the expression was studied after seven days of inoculation. *PvOSCA* genes are observed to be mostly downregulated when infected with rhizobial and mycorrhizal fungi (Fig. 8c and Supplementary Table 7d).

## Discussion

OSCA was first identified in *Arabidopsis thaliana* as an osmosensor that mediates the upheaval of hyperosmolality-induced cellular calcium concentration^15^. The discovery of OSCA has considerably expanded the understanding of plants' molecular mechanisms involved in osmotic stress tolerance. It is designated to be a mechanically activated ion channel^4^ which controls the Ca^2**+**^ level in the cytosol by activation of influx conductance. Homologs of *OSCA1* are found to mediate the closure of stomatal pores during immunological responses^22^. OSCA might have crucial roles in recognizing the exogenous and endogenous osmotic changes that regulate plant growth and development.

OSCA is a multi-member family with 15 identified members in *A. thaliana*^15^, 11 in rice^6^, 16 in Pear^7^, and 12 in *Zea mays*^1^ genome respectively. However, no detailed comparative analysis of the OSCA gene family has been conducted in leguminous plants. The current study has been conducted on the *OSCA* gene family in the four legume plant species namely *Cicer arietinum*, *Cajanus cajan*, *Vigna radiata,* and *Phaseolus vulgaris*.

In our study, we identified 51 *OSCA* genes in the 4 legumes, 12 in *C. arietinum* and 13 each in *C. cajan, V. radiata* and *P. vulgaris*. They exhibit a nearly consistent average molecular weight of 85 kDa, and an average amino acid length of 747, except CaOSCA2.1 displays a lower molecular weight and smaller length, which is supported by the presence of a truncated RSN1_TM domain, as discussed earlier.

All the 51 OSCAs have been systematically analysed to check their phylogenetic relationship with *A. thaliana* and *G. max.* The consistency of the number of OSCAs across the genomes of the four legume species suggested a relatively stable evolutionary history. All 86 (15 in *Arabidopsis,* 20 in soybean*,* 12 in *Cicer,* and 13 in each of *Cajanus, Vigna* and *Phaseolus*) OSCAs have been clustered in 4 distinct clades. In *Cicer*, members in clade I are non-identical with the other three legumes, it has four members in Clade I in place of five members as exhibited by the other three legumes. This might indicate an event of gene loss or deletion. Clade III and clade IV are notably smaller. In spite of that, each of these two clades shares distinctly similar properties and functions within themselves, which indicates they have some evolutionary significance.

The protein structure of OSCA revealed the presence of three well-defined domains. RSN1_TM-an N-terminal transmembrane domain which is associated with Golgi transport during late exocytosis, PHM7_cyt which is the cytosolic domain of the 10TM region having putative phosphate transporter functions, and RSN1_7TM which is a calcium-dependent channel and is predicted to be a 7TM region having putative phosphate function. This RSN1_7TM region present in the C-terminal is predicted to be the DUF-221 domain^7^. The entire OSCA family is characterized by the presence of the highly conserved DUF221 region, which functions as an osmotic-sensing calcium channel^6,17^. According to InterPro and Pfam, DUF221 represents the seven transmembrane domain regions of the calcium-dependent channel and is homologous to domains in anoctamin/TMEM16 channel^5,23^, which are calcium-activated chloride channel (CaCC) component, and salt chemosensation transmembrane channel-like (TMC) proteins in *C. elegans*^24^ or mechanosensitive TMCs in hair cells of the mammalian inner ear^25^. The PHM7_cyt domain is the predicted cytosolic domain of integral membrane proteins also present in yeast and humans^26^, and is probably distantly homologous with RNA-binding proteins of the RRM family. The OSCA proteins are characterised by the presence of distinct transmembrane helices. All the 51 OSCA proteins in the present study consist of 6–11 transmembrane helices. Similar instances have been observed in the OSCA family in rice (6–10 transmembrane helices) and pear (10–11 transmembrane helices)^6,7^. Various studies reveal that the manifold arrangement of the intron–exon results in the evolution of multifarious gene families by any of the three methods-exon/intron gain/loss, exonisation/pseudoexonisation, and insertion/deletion^7^. The genetic construction of *OSCA* genes is highly consistent with respect to their exon–intron design, indicating that they were well-conserved during evolution. There is a distinct presence of exon gain and loss events in the gene structure of the clade I and II members. These events have dominated the evolution of the *OSCA* genes. *CaOSCA2.3* contains a large intron, suggesting a seemingly more complex function. A similar instance has been noticed in the OSCA family in *Gossypium hirsutum* where *GhOSCA1.6* reveals a structure different from all the other *GhOSCA* genes, characterised by a large intron^14^. In addition, *OSCA4.1* in all four plant species are distinguishably intron-poor structures with no intron and only one exon, which might suggest a difference in their function from the *OSCA* genes in the rest of the three clades. Though different intronless genes are noticeable in eukaryotes, the lack of introns is a prokaryotic feature. There is no sufficient insight into the exact functions of intronless eukaryotic genes. Previous studies have shown that intronless genes first evolved in early land plant evolution and indicated the correlation between intron-loss and development, metabolism, and housekeeping functions. Hence the intronless *OSCA* genes of clade IV possibly indicate early evolution and are actively involved in plant metabolism and development^27,28^.

Unique motif structures in all 51 OSCA proteins show the similarity in motif structure and arrangement in Clade I, II, and III, suggesting a functional similarity. The exceptional motif arrangement possessed by CaOSCA2.1 is supported by its domain structure, which portrays a relatively shorter RSN1_TM domain. Interestingly, Clade IV in each of the four species contains noticeably different motif composition and structure than the rest of the three clades but shares a consistent motif arrangement. This suggests evolutionary significance in the OSCA proteins present in Clade IV. Therefore, with respect to high molecular weight (> 90 kDa), mildly acidic isoelectric point (≈ 6–7), intron-poor gene structure, presence of two DUF221 domains and distinctly different motif assembly, the clade IV *OSCA* genes are unique in nature than the rest of the three clades. Hence, they might have differences in function from the other *OSCA* genes. This also indicates the diversification of the *OSCA* gene family.

Chromosome structure and arrangement of genes reveal the presence of segmental duplication in the OSCA family in the four legume species. Our results have suggested that both intraspecific and interspecific gene duplication events occur. There are pieces of evidence of both tandem and segmental duplication events, along with occurrences of dispersed gene duplication. This indicates that all three types of gene duplications have played a major role in the diversification and expansion of the *OSCA* gene family. The presence of significant numbers of collinear genes between soybean and the four legumes in our study further strengthens the fact that gene duplication played a crucial role in the evolution of the *OSCA* gene family. Additionally, it is possible that they generated novel gene functions.

Ka/Ks ratio is the ratio of mutations utilized to determine the selection pressure on any protein or gene. Ideally, Ka/Ks > 1 signifies accelerated evolution with positive selection; conversely, Ka/Ks < 1 signifies purifying selection^18^. All the duplicated pairs of genes display a value of < 1, which signifies all four plants have undergone purifying selection, i.e., there has been a selective removal of deleterious alleles in the process of evolution^29^.

One of the significant players in the stress response activity of OSCA is the cis-regulatory elements. Cis-regulatory elements function as molecular switches which regulate gene expression. Transcription factors and cis-regulatory elements combine together to play a major role in establishing the crosstalk among several stress signalling pathways. 16 cis-regulatory elements are identified in the four plant species in the current study, which are expressed in the *OSCA* genes. G-box is cis-acting, ubiquitous, DNA- binding elements^30^, also known to play an important role in managing biotic and abiotic stress. It also acts as a positive regulator of senescence^19^. GCN4 plays a central role in controlling endosperm-specific expression^31^ and regulates seed-specific expression of the genes^32^. Elements like ERE, TCA-element, and TGA-element regulate morphological traits. ERE is an Ethylene responsive element, TCA is salicylic acid-responsive element, TGA is an auxin responsiveness element which is also found to have a regulatory function during iron deficiency^33,34^. Moreover, TGA is a class of leucine zipper transcription factors that function in plant’s defence mechanism by binding with NPR1 monomers during pathogen attack^35^. ABRE is an ABA-responsive element that response to various adverse environmental conditions such as drought, high salt, and cold or freezing conditions^36^. WUN motif was found to be a wound-responsive element, also detected in *Brassica oleracea*. STRE and TC-rich repeats are stress-responsive elements. LTR is a low-temperature responsive element. The presence of stress-responsive elements like G-box and STRE, cold-responsive elements like LTR and ABA-responsive (ABRE) cis-regulatory elements in most of the OSCA promoter regions suggests a possibility of OSCA transcription being activated by ABA and multiple abiotic stresses. Since both stress-sensitive (STRE, TC-rich repeats) and hormone-responsive (ERE etc.) cis-regulatory elements are detected in their promoter region, it indicates that OSCAs could mediate the crosstalk of stress and hormone signalling pathways. Thus, OSCAs are potential candidates in hormone-mediated abiotic stress signalling in the four legume plant species^37^. The abundance of STRE elements in the promoter of *CcOSCA, VrOSCA,* and *PvOSCA* genes supports their upregulation when subjected to different stress conditions. In contrast, the rare occurrence of STRE elements in *CaOSCA* genes supports their mostly downregulated activity. Expression analysis studies show that *CaOSCA2.1* is involved in cold stress, which is evident from the presence of LTR in its promoter. In addition, most OSCA genes are highly expressed during the growth and development stages of the legumes in the study, which is supported by ARE and Box 4 elements abundant in their promoter region.

The Gene Ontology (GO) analysis, used to classify genes and their products, exhibited cation membrane transport as the most distinct function of the *OSCA* genes. In general, OSCAs are associated with cation transport across the membrane, which is activated by calcium, as evident from previous research works^6^, further strengthened by the GO-analysis results in the current study. The study shows that the members of clade IV regulate unique biological processes and possess the most diverse range of molecular functions. Intriguingly, molecular functions like pyridoxal phosphate and hydrolase activities are more common in clade II than in any other clade. Based on the GO terms obtained from annotation, the *OSCA* gene family will likely be involved in cellular metabolic pathways.

An adaptation pattern for survival is activated when plants are exposed to unfavourable environmental conditions that alter their gene expression from a programmed pattern for normal development into an adaptive pattern that allows them to survive. Gene expression is a direct display of the functionality of a particular gene. A thorough investigation of the *OSCA* genes exhibits their role in the growth and development of plants and various biotic and abiotic stress conditions. To assess the expression profile of the *OSCA* genes, transcriptome data of the *CaOSCAs, CcOSCAs, VrOSCAs,* and *PvOSCAs* were explored. Investigating the expression profile of OSCA in various developmental stages of *Cicer*, *Cajanus,* and *Phaseolus*, it is evident that OSCA plays a major role in the growth and development of these plants at different stages of development. Most *OSCA* genes display highly upregulated expression in various growth stages, with certain exceptions in all three plant species. Interestingly, Clade II genes, especially *OSCA2.3* and *-2.5*, were mostly found to be downregulated in the different developmental stages; hence they might not have a significant role in plant development.

*OSCA* genes reveal a multi-dimensional role in abiotic stress. Notably, the degree of upregulation of *CaOSCA* genes when subjected to desiccation, salinity and cold stresses is more in the shoot than in the root. *CaOSCA2.6* exhibits high expression in all three stresses, and qRT-PCR results validate its role in salt stress. Experimental validation through qRT-PCR shows *CaOSCA2.1* to have the highest degree of expression under desiccation stress. The role of *CaOSCA2.1* is also supported by in silico study where it is upregulated in shoots under drought stress. *CaOSCA2.3 and -2.6* are upregulated in salt stress, as observed through computational and experimental methods. In silico results also indicate that *VrOSCA* genes might have a prominent role in desiccation stress. In *C. cajan,* most *CcOSCA* genes are upregulated in salt-susceptible and tolerant genotype variants in both shoot and root. However, susceptible shoot displays a greater number of upregulated genes. In *P. vulgaris*, more OSCA genes are induced in leaf and root tissues in salinity-susceptible genotype TR43477 than in salinity-tolerant genotype Ispir. From this observation, it is evident that *OSCA* genes might play a complicated role in salinity tolerance mechanisms which is yet to be explored. The comparative assessment shows that *PvOSCA* genes have a greater transcriptomic abundance than *CaOSCA* and *CcOSCA* genes with respect to salinity stress.

Additionally, the expression profile of *OSCA* genes was studied for drought treatment in *P. vulgaris*. Most *PvOSCA*s are upregulated in both *BAT 477* and *Perola* genotypes with a few exceptions. But the degree of upregulation varies, and the leaf tissues are more commonly upregulated than the root in both genotypes. The upregulation of *PvOSCA2.5, PvOSCA2.6, PvOSCA2.1* in drought-tolerant genotypes indicates that they might play a crucial role in drought tolerance in *P. vulgaris.* By close investigation of the *CaOSCA* and *PvOSCA* genes, it is apparent that OSCAs are closely associated with drought stress regulation in plants.

Moreover, the expression of *OSCA* genes correlated with various biotic stress has been demonstrated in *C. arietinum, V. radiata* and *P. vulgaris* in a tissue-specific manner. The upregulated expression of *CaOSCA2.5* and in *CaOSCA1.4* in resistant genotypes of *Cicer* subjected to *Ascochyta rabiei*, a common blight fungus infection indicates their possible role in biotic stress regulation. In *V. radiata,* only two out of 13 genes showed noticeable expression in *Fusarium* infection; hence it can be inferred that the *VrOSCA* genes might not play a significant role when subjected to biotic stress. Also, in *P. vulgaris, PvOSCA* genes are commonly downregulated when subjected to rhizobial and mycorrhizal fungi infection*.* But the *PvOSCA* are commonly upregulated in the cumulative effect of abiotic (drought) and biotic (AMF) stress. Hence, these results suggest that the *OSCA* genes might not significantly regulate biotic stress in plants, but there is a scope for future research in this area. It is evident that the *OSCA* gene family plays a crucial role in regulating developmental stages and stress tolerance in various vegetative tissues in plants. The notable changes observed in the OSCAs of the four legumes regarding their general features and gene expression are listed in Table 1.

**Table 1:** Unique changes in the *OSCA* gene family in four legumes viz *C. arietinum, C. cajan, V. radiata, P. vulgaris*.

Characteristic attributes	Unique changes/features of *CaOSCA*, *CcOSCA*, *VrOSCA*, and *PvOSCA*
Phylogenetic classification	Four members: clade I (*C. arietinum*) Five members: clade 1 (*C. cajan, V. radiata, P. vulgaris*)
Gene structure	Exon gain: *CaOSCA2.2*, *CcOSCA2.1, CcOSCA2.2, VrOsCA2.1, VrOsCA2.2 and PvOSCA2.1* Intronless structures: Clade IV proteins Long intronic structure: *CcOSCA2.3*
Essential domains	RSN1_TM, PHM7_cyt and RSN1_7TM: all clades Two RSN1_7TM (DUF221) domains: clade IV Truncated RSN1_TM domain: CaOSCA2.1
Transmembrane helices	6–11 transmembrane helices
Three-dimensional structure	Each OSCA protein: 11 distinct helices
Subcellular localization	Plasma membrane
Motif assembly	Clade I and II: Similar motif assembly Motif 13: part of essential domain RSN1_TM Motif 6: part of essential domain RSN1_7TM (DUF221)
Gene duplication types	Whole genome/segmental, tandem, and dispersed Purifying selection pressure in all the duplicated gene pairs
Promoter analysis	Stress-responsive, hormone-responsive, and Growth and metabolism related cis-elements
Biological processes and molecular function	Calcium-activated cation channel activity Cation transmembrane transport
Growth and development	~ 50% OSCA genes
Abiotic stress regulation	Drought stress: *CaOSAC2.1, PvOSCA2.5, PvOSCA2.6, PvOSCA2.1* Salt stress: *CaOSAC2.6, CcOSCA2.6, CcOSCA3.1*

## Materials and method

### Identification of OSCA proteins in legumes

To identify the OSCA proteins in the target legume species, protein sequences of *Cicer arietinum*, *Cajanus cajan*, *Vigna radiata*, and *Phaseolus vulgaris* were downloaded from NCBI (https://www.ncbi.nlm.nih.gov/refseq/). Formerly characterized OSCA protein sequences of *Arabidopsis thaliana* and *Oryza sativa* were retrieved from UniProt (Swiss-Prot) (https://www.uniprot.org), and homology search was performed using the BLAST tool (at *E*-value of 1e−5) against the proteome of all four aforementioned legume plants. Significant hits were selected based on ≥ 50% identity and ≥ 100 amino acid length alignment^38,39^. After that, all four sets of putative candidates were screened using CD-hit^40^, followed by manual curation to remove redundant sequences. The presence of essential domains was then verified in the resulting sequences using a standalone version of InterProScan^41^. Finally, 13 members were confirmed in each of *C. cajan*, *V. radiata*, and *P. vulgaris,* and 12 members in *C. arietinum*. The different attributes of the identified OSCA gene families, such as gene ID, protein ID, CDS, size of amino acid, and chromosomal coordinates, were extracted from the NCBI web server.

### Phylogenetic analysis

The evolutionary relationship between the OSCAs of *Cicer arietinum, Cajanus cajan, Vigna radiata, Phaseolus vulgaris*, *Arabidopsis thaliana*, and *Glycine max*^42^ was investigated through Multiple Sequence Alignment (MSA) of the amino acid sequences of OSCAs of all the six land plant species by using CLUSTALW^43^ in MEGA version 11.0.13^44^. The parameters for constructing the phylogenetic tree are as follows, (1) Scope: All selected taxa, (2) Statistical method: Neighbor-joining, (3) Test of Phylogeny: Bootstrap method, (4) The number of bootstrap replicates: 1000, (5) Substitutions type: Amino acid, (6) Model: Poisson model, (7) Rates among sites: Uniform rates, (8) The pattern among lineages: Same (homogenous), (9) Gaps/missing data treatment: Pairwise deletion. With the iTOL^45^ web server, it was possible to achieve an improved visualization of the phylogenetic tree.

### Gene structures, motif organization, and domain prediction

The Gene Structure Display Server (GSDS)^46^ program was used to analyse the exon, intron, and untranslated region organization of the OSCA genes by comparing CDS sequences with corresponding genomic DNA sequences. MEME (Multiple Expression motifs for Motif Elicitation) webserver^47^ was used for the identification and schematic representation of motif organization in OSCA proteins. This tool was used at the following parameters: site distribution–zero or one occurrence per sequence (zoop); motif discovery mode-classic; optimum motif length was between 6 and 200; and the top 15 most enriched motifs were selected based on the lowest e-values^48^. Identification of domains in OSCAs proteins was conducted by InterProScan^41^, Pfam^49^, SMART^50^, and Illustrator of Biological samples (IBS) webserver were used for pictorial representation of the essential domains^51^.

### Gene nomenclature, chromosomal location, gene duplication, and promoter analysis

The nomenclature of *OSCA* genes in each of the four plant species was assigned according to their closest orthologs of *Arabidopsis thaliana OSCA* genes in the phylogenetic tree. Gene and protein ID and information on chromosomal coordinates were extracted from NCBI. MG2C tool^52^ was used to construct the chromosome maps for each four plant species, which display the position of each gene on their respective chromosomes. To search for putative cis-acting elements and their possible involvement in stress regulation and plant development, the 2000 bp upstream sequences as promoter regions were compiled from NCBI and used as input to the PlantCARE database^53^.

### Gene duplication and ka/ks value calculation

To search for all the duplicated gene pairs within the *C. arietinum, C. cajan, V. radiata,* and *P. vulgaris* genome, the protein sequences of the mentioned plant species were used to run the all-versus-all local BLASTP with parameters of E-value 1e-5, max target sequences 5, and m6 format output. MCScanX software is used to obtain data on collinear pairs of genes^54^. From this information, we utilized CIRCOS software package to map the intraspecific, and interspecific duplicate gene pairs present in each of the four plant species^55^. Each 51 *OSCA* gene is classified according to its duplication types using the duplicate_gene_classifier script in MCScanX. The selection pressure of the *OSCA* genes was evaluated by the Ka and Ks values^18^. PAL2NAL web server was utilized to determine the rate ratio of synonymous (Ks) and nonsynonymous (Ka) substitutions of duplicated *OSCA* genes^38,56^.

To enhance the systematic of the current research, collinearity analysis was carried out between previously characterized OSCAs of another economically important legume crop *G. max* (Soybean)^42^, and the four main legumes in our study. MCScanX and CIRCOS software were utilized to detect and visually represent the interspecific collinear gene pairs between the five aforementioned legumes.

### Subcellular localization and physicochemical properties of OSCA proteins

CELLO^57^ is used to predict the subcellular localization of all the OSCAs identified in all four plant species. The subcellular localization of the OSCAs was illustrated by the Biorender software package (https://biorender.com/). The molecular weight (MW) and isoelectric point (pI) of OSCA proteins were calculated using the online tool 'Compute pI/MW' from Expasy^58^.

### Prediction of protein tertiary structure

The tertiary structures of all OSCA proteins were predicted with the Phyre2 web portal (http://www.sbg.bio.ic.ac.uk/Phyre2)^59^. The transmembrane helices present in OSCA proteins were determined by TMHMM 2.0 tool^60^.

### Gene ontology analysis

Gene ontology (GO) analysis of the 51 OSCA proteins was performed using PANNZER (Protein ANNotation with Z-scoRE) server^61^.

### Expression analysis using RNA-seq data

The genome-wide expression profiles of OSCA genes for different abiotic and biotic stresses and developmental stages were yielded through RNA-seq data from the Sequence Read Archive (SRA) of NCBI. Raw RNA-seq reads downloaded from SRA of NCBI and thereafter were processed by FASTP to remove the adapters, poly-N, short and low-quality reads^62^. From the NCBI genome web server, the reference genomes of *C. arietinum, C. cajan, V. radiata,* and *P. vulgaris* were downloaded. Each reference genome's index was created using the HISAT2 tool^63^, and filtered reads were mapped onto the corresponding genome index. The alignments were assembled into potential transcripts using StringTie^64^ software package, and fragments per kilobase of transcript per million reads (FPKM) values were used to calculate the transcript abundance. To calculate the differential expression, fold change expression was calculated by the ratio of the average FPKM of both test samples and control samples. The ‘Pheatmap’ package of R was used to generate the heatmaps of the expression data using the logarithm of normalized expression values for the developmental stages study and the logarithm of fold change for the other studies.

### Plant material, growth conditions, and stress treatment

Desi Chickpea (*Cicer arietinum* L. genotype ICC4958) seeds were grown as described^65^. The seedlings were grown in the sterile mixture (1:1) of agro-peat and vermiculite in plastic pots at 22 °C in a culture room with a light–dark cycle of 14 h/10 h. For drought treatment, 21 day-old seedlings were removed from the pot and transferred to folds of tissue paper for five hours. For salinity stress, seedlings were transferred to a beaker containing 150 mM NaCl solution at 22 °C. For cold treatment, the potted seedlings were kept at 4 °C. The control seedlings were kept in plastic pots at 22 °C. Tissues were collected from the stressed and control seedlings after 5 h of the treatment. Three independent biological replicates of each tissue sample were harvested, frozen in liquid nitrogen, and stored at − 80 °C until further use.

### Total RNA isolation and cDNA preparation, quantitative real-time PCR validation

Total RNA was isolated from 100 mg of tissue using the RNeasy Plant Mini Kit (Qiagen) according to the manufacturer's instructions. The quality and quantity of RNA samples were assessed using Nanodrop Spectrophotometer (NanoDrop Technologies) and Bioanalyzer (Agilent technologies). Only the RNA samples with a 260/280 ratio from 1.9 to 2.1, a 260/230 ratio from 2.0 to 2.4, and RNA integrity number (RIN) of more than 7.0 were used for the analysis. DNase treatment was applied to 5 μg of total RNA using RNase-Free DNase Set (Qiagen) following the manufacturer's instructions. The cDNA was synthesized from 2 µg RNA using the Verso cDNA synthesis kit (Thermo Scientific™). The real-time PCR assay was performed with the reaction mixture (10 μl) consisting of 5 μl of 2 X SyBr green master mix (Applied Biosystems™), 10 μM of each primer, and 100 ng of cDNA as the template. The reaction was performed using Applied Biosystems™ qPCR with temperature profiling set at 95 °C for 2 min followed by 40 cycles at 95 °C for 15 s and 60 °C for 1 min. All the reactions were carried out at least in three biological and three technical replicates. The obtained data were evaluated using the 2^−△△Ct^ method to determine the relative expression level^66^.

### Statistical analysis

All experimental data were expressed as the mean with standard deviation (mean ± SD) of three independent biological replicates. A test of statistical significance using a comparison among the means of control and stressed plants was conducted using one-way analysis of variance (ANOVA) followed by Student’s t-test at *P*-value < 0.05. The *P*-values < 0.05 were considered statistically significant.

## Conclusion

The OSCA acts as an osmosensor in plants and was found to have regulatory roles in drought stress. This study provides a systematic and comparative analysis of OSCA genes in four economically important legumes. A total of 13 *OSCA* genes were identified in *C. cajan, V. radiata, P. vulgaris;* and 12 *OSCA* genes were identified in *C. arietinum.* The results shed light on structural and physicochemical attributes of the *OSCA* gene family, which shows that this gene family is evolutionarily conserved. The results provide a novel insight into the role of *OSCA* genes in growth and development and in the mediation of biotic and abiotic stress. *CaOSCA2.1, CaOSCA2.6,* and *CaOSCA1.1* can be candidate genes in drought, salinity, and biotic stress, respectively. *CcOSCA3.1* and *CcOSCA2*.*6* can act as candidate genes in salinity stress. Owing to their overexpression in both biotic and abiotic stresses, *VrOSCA3.1* can be deemed as a candidate gene. *PvOSCA2.1* can be regarded as a candidate gene for cumulative stress factors. These candidate genes can be further explored and used in genetic engineering techniques to devise stress-tolerant crops. Our study will improve the understanding of the regulatory mechanism of *CaOSCA, CcOSCA, VrOSCA,* and *PvOSCA* genes in multiple abiotic stresses and in plant-pathogen interplay. Additional research might be conducted to determine the possible targets of the *OSCA* genes in the four legumes and identify the OSCA genes most responsive to a particular stress element.

## Supplementary Information

Supplementary Figures.

Supplementary Table 1.

Supplementary Table 2.

Supplementary Table 3.

Supplementary Table 4.

Supplementary Table 5.

Supplementary Table 6.

Supplementary Table 7.

## Data Availability

RNA-Seq data used in this study is available at the Short Read Archive (SRA) of NCBI with BioProject PRJNA413872, PRJNA479940, PRJNA328302, PRJNA232700, PRJNA436616, PRJNA344973, PRJNA354681, PRJNA382795, PRJNA343064, PRJNA327304, PRJNA715596, PRJNA656794, PRJNA311998, PRJNA327176, PRJNA482464, and PRJNA221782.
