# Supplementary material for: Genome-wide characterization and comparative analysis of the OSCA gene family and identification of its potential stress-responsive members in legumes
Source: Sci Rep. 2023 Apr 11;13:5914. doi: 10.1038/s41598-023-33226-8 (PMC10090146; doi:10.1038/s41598-023-33226-8)

**Supplementary Figures**

**Figure S1:** Three-dimensional protein structure of OSCA protein monomers in legumes elucidating the 11 conserved 11 helices, the N-terminal, and C-terminal regions in representative members of Clade I, II, III, and IV. CaOSCA2.1 clearly shows a much shorter helix 2 than others.


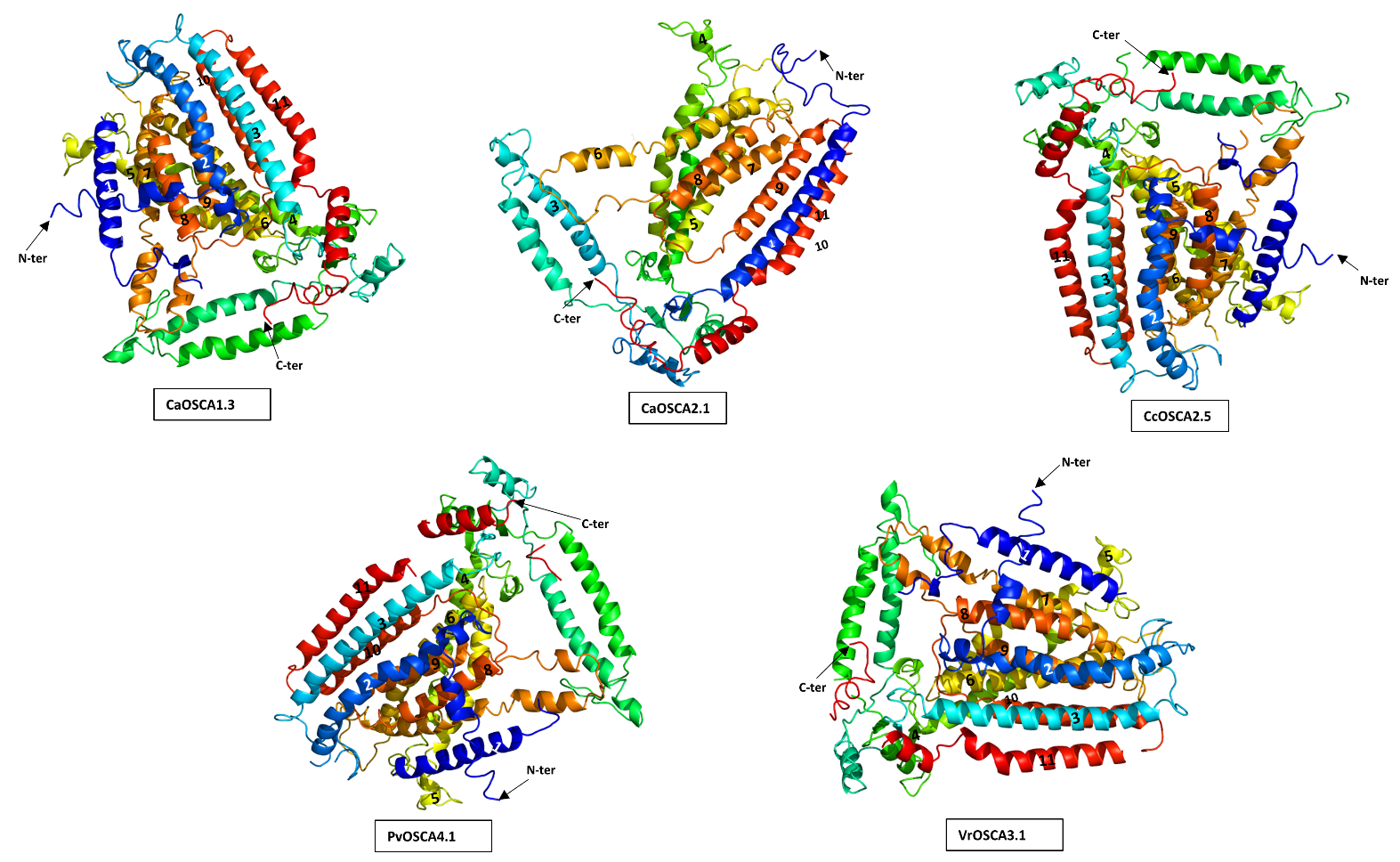


**Figure S2:** Subcellular localization of CaOSCA, CcOSCA, VrOSCA and PvOSCA proteins.


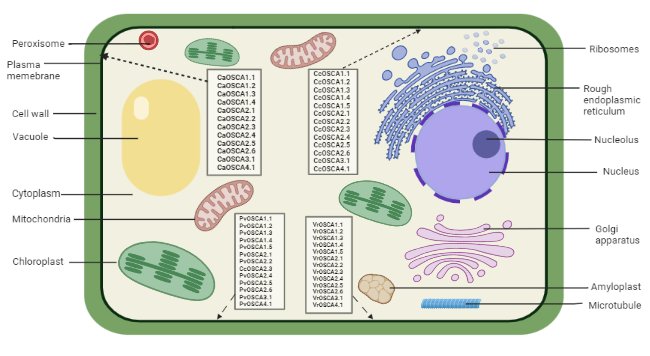


**Figure S3:** Transmembrane domains present in the OSCA protein (indicated in red).

**
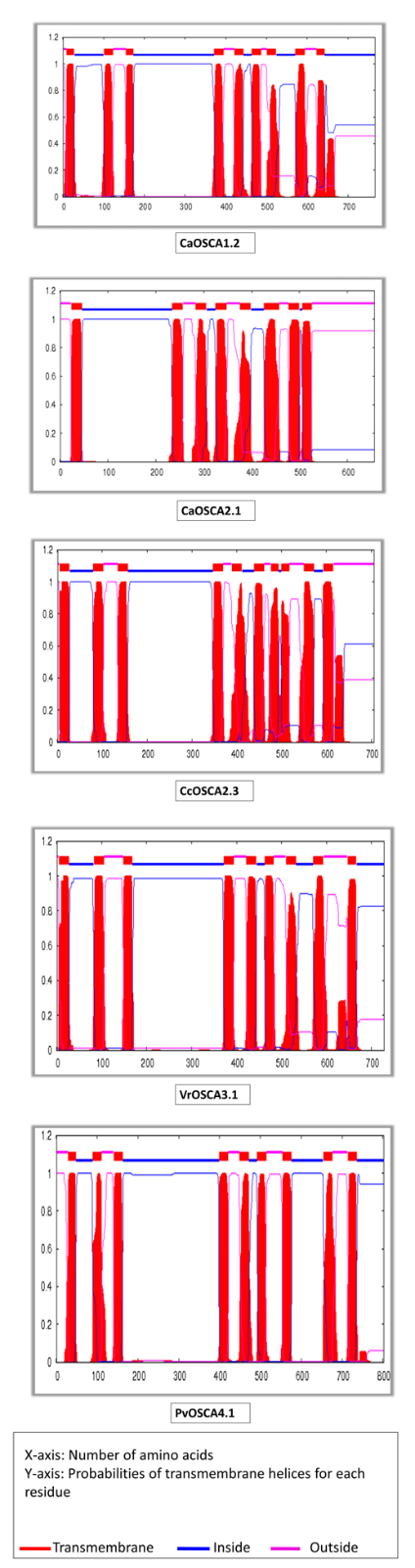
**

**Figures S4(a-c):** Unique motif structures present in OSCA proteins shown in different colours. The amino acid sequence of each motif is also shown (a. Motif logos, b. Motif patterns, c. Motif sequences).

Motif logos

|  | **E-value** | **Sites** | **Width** |
| --- | --- | --- | --- |
| 1 | 2.3e-1892 | 16 | 159 |
| 2 | 3.4e-1783 | 51 | 58 |
| 3 | 1.7e-1442 | 20 | 120 |
| 4 | 1.5e-772 | 46 | 38 |
| 5 | 7.9e-1062 | 47 | 44 |
| 6 | 1.7e-1008 | 37 | 43 |
| 7 | 6.0e-1063 | 37 | 51 |
| 8 | 1.1e-1113 | 19 | 85 |
| 9 | 1.6e-707 | 47 | 29 |
| 10 | 2.7e-676 | 31 | 50 |
| 11 | 2.4e-549 | 46 | 23 |
| 12 | 2.0e-611 | 34 | 41 |
| 13 | 6.8e-430 | 51 | 21 |
| 14 | 9.2e-364 | 4 | 200 |
| 15 | 2.3e-237 | 37 | 21 |

| 1 |
| --- |
| 2 |
| 3 |
| 4 |
| 5 |
| 6 |
| 7 |
| 8 |
| 9 |
| 10 |
| 11 |
| 12 |
| 13 |
| 14 |
| 15 |


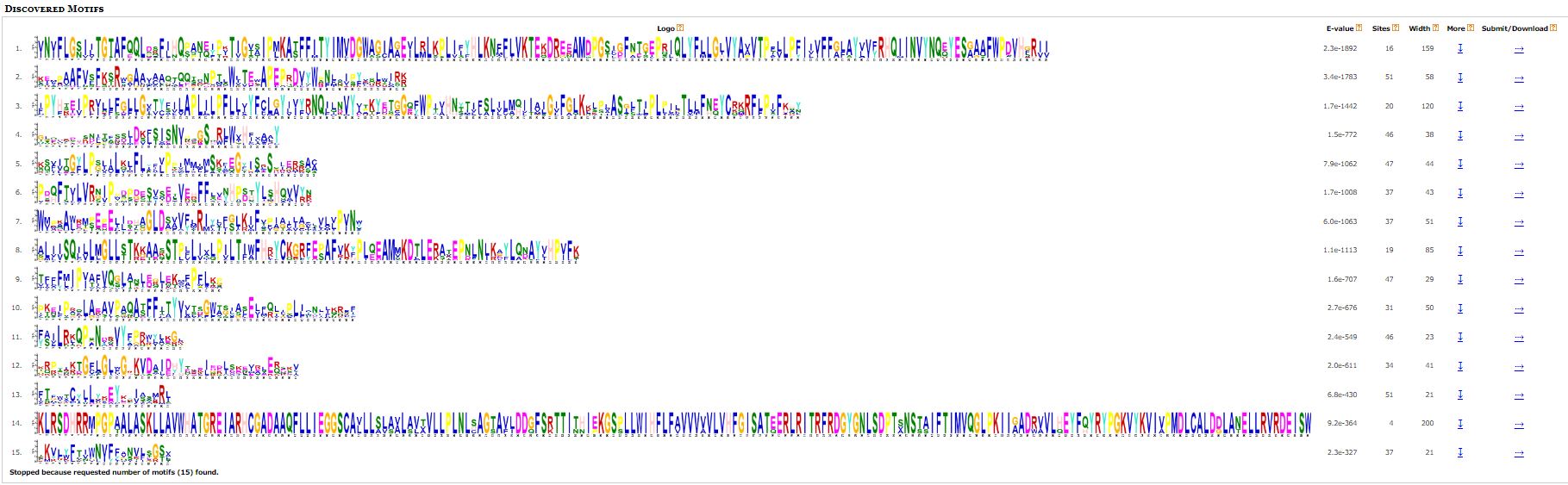


**(a)**


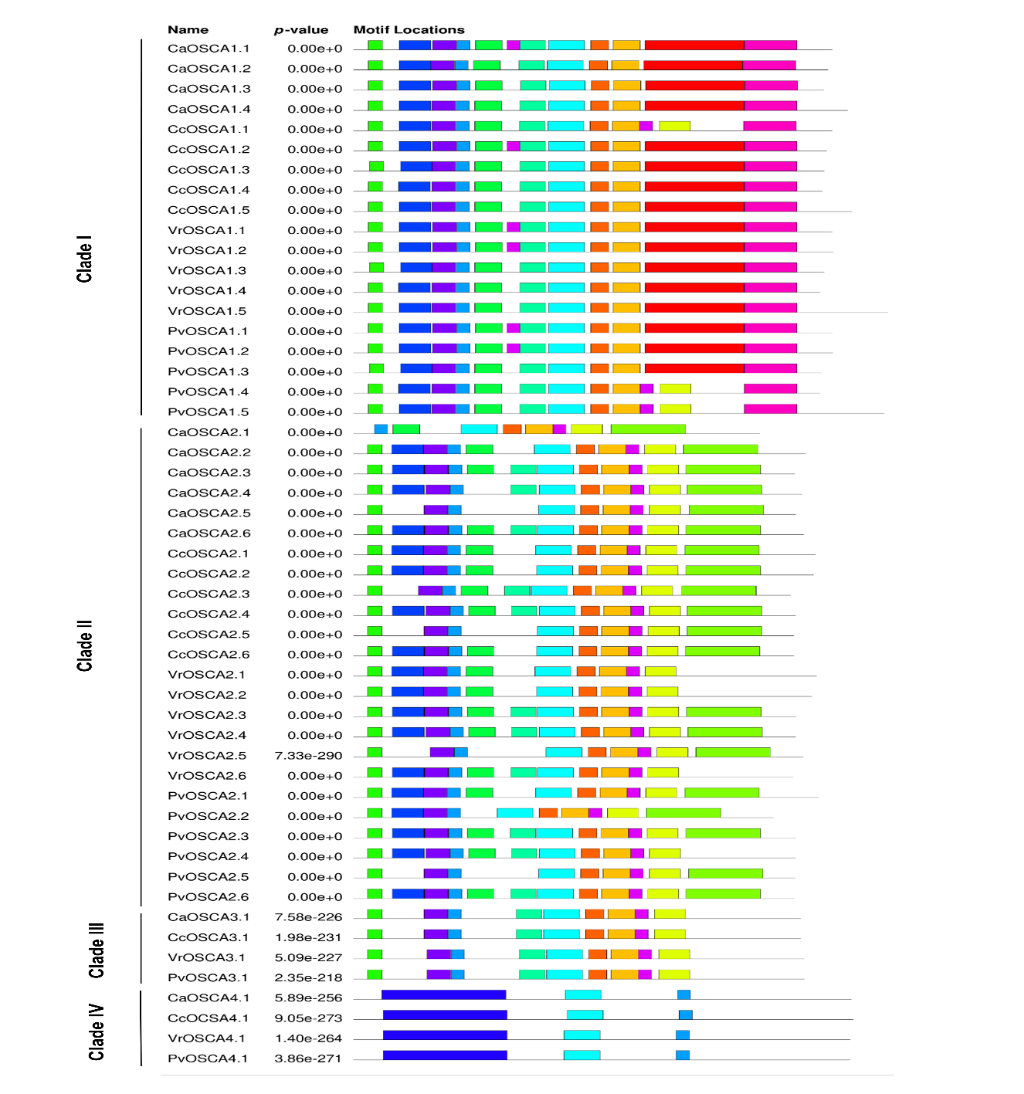


**(b)**


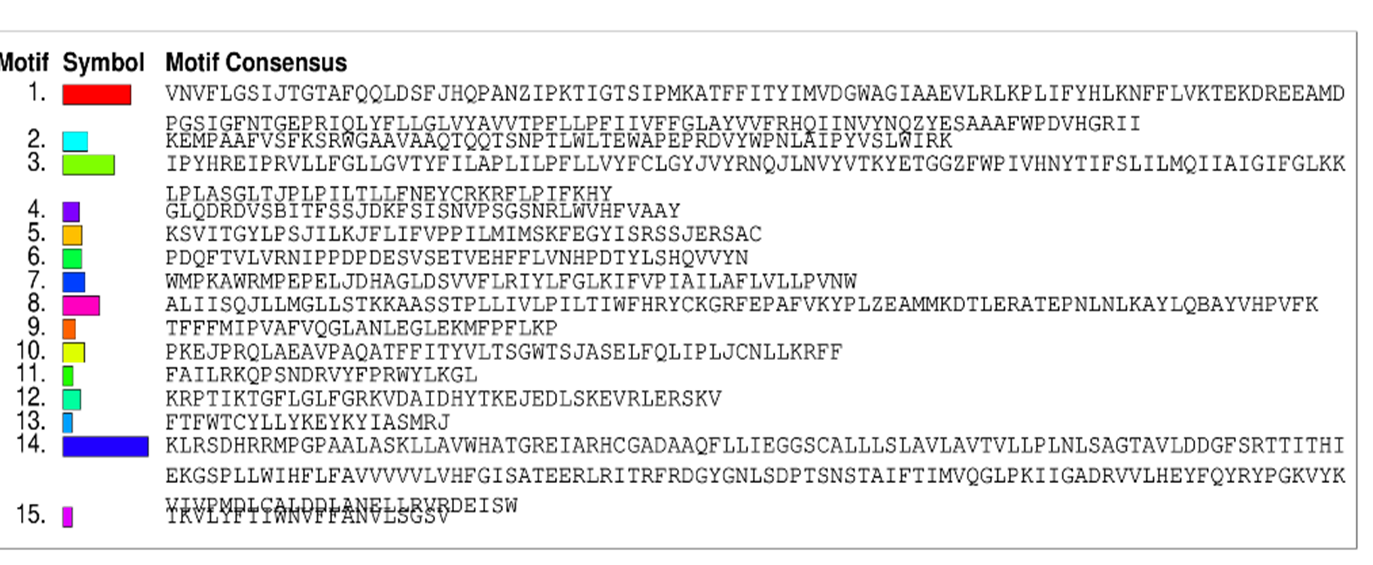


**(c)**

**Figure S5:** Chromosome map showing the location of OSCA genes in each legume species on their respective chromosomes

**
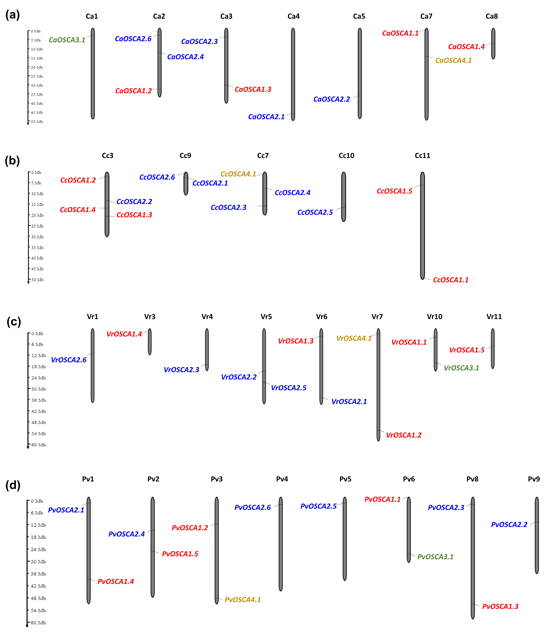
**


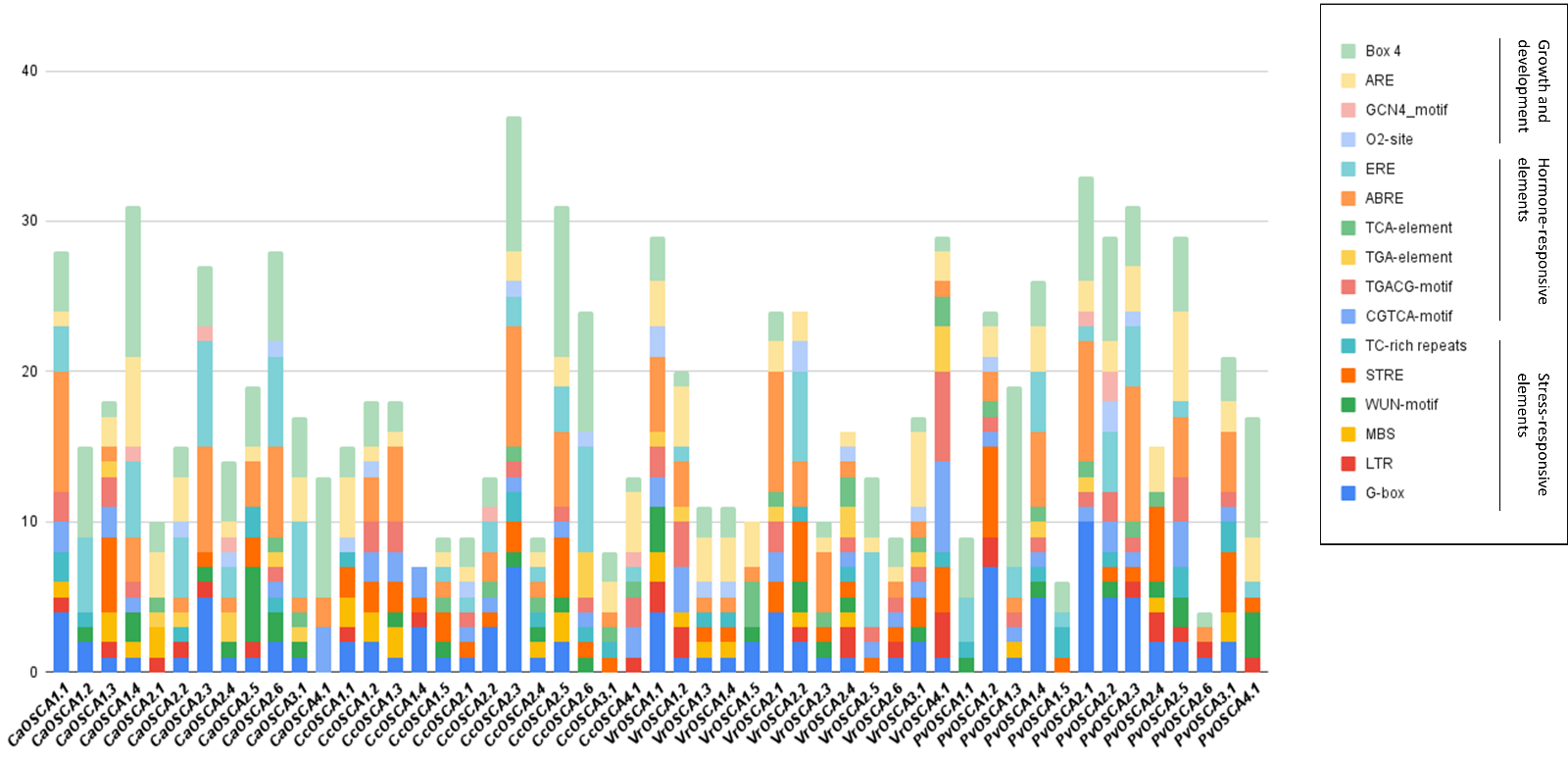
**Figure S6:** Different cis-regulatory elements in the 2 kb upstream region of *OSCA* genes are illustrated by different colours in the bar chart. The X-axis represents the name of genes and Y-axis represents the number of cis-elements in each promoter. The names of cis-regulatory elements are mentioned along the graph.

**Figure S7:**  The GO enrichment of OSCA is depicted in the pie chart showing, (a) Biological processes, (b) molecular functions, and (c) cellular components


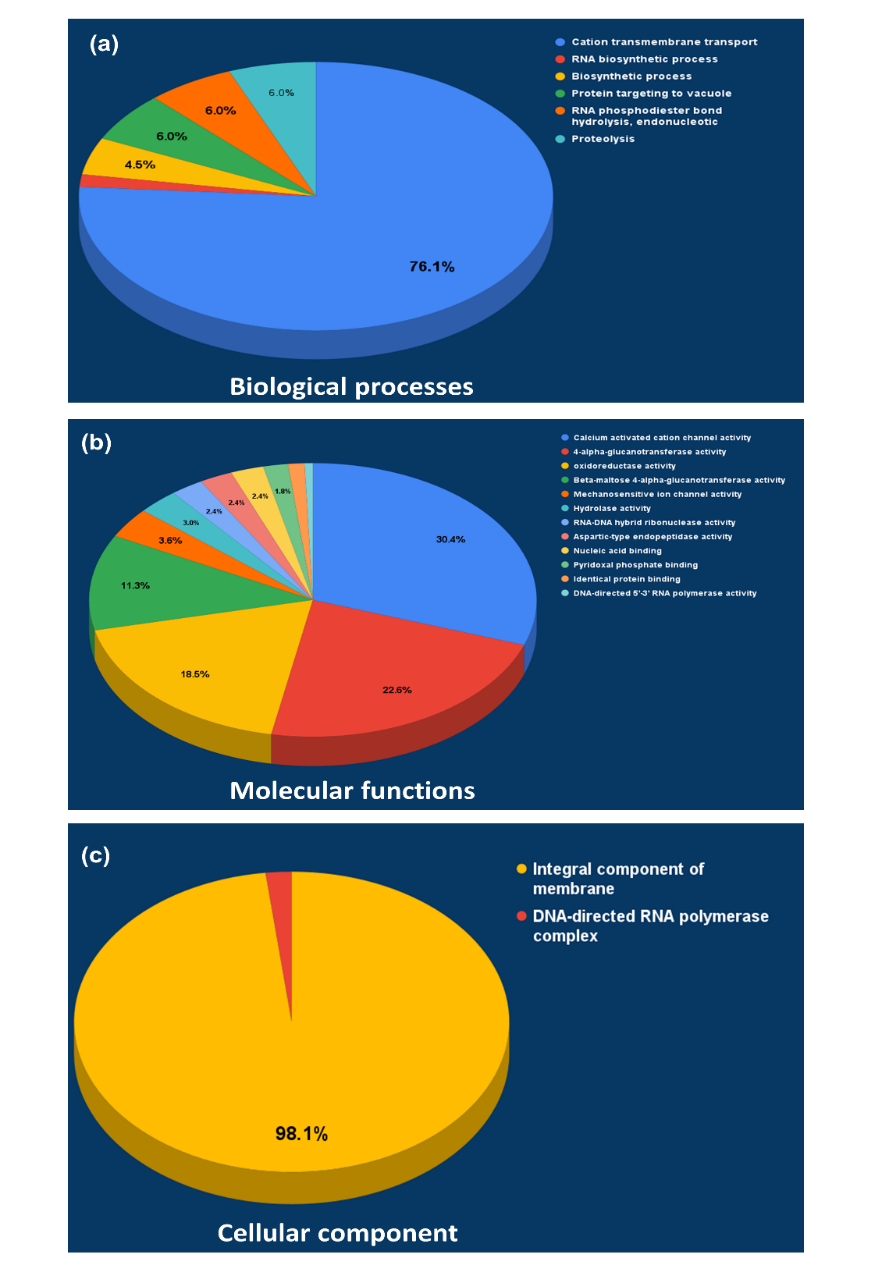

Supplement: Supplementary file 1 — Supplementary Figures. [file 41598_2023_33226_MOESM1_ESM.docx]
